# Identification of HPr kinase/phosphorylase inhibitors: novel antimicrobials against resistant *Enterococcus faecalis*

**DOI:** 10.1007/s10822-022-00461-6

**Published:** 2022-07-09

**Authors:** Sandeep Kumar, Rajendra Bhadane, Shruti Shandilya, Outi M. H. Salo-Ahen, Suman Kapila

**Affiliations:** 1 Animal Biochemistry Division, National Dairy Research Institute, Karnal, Haryana India; 2 Structural Bioinformatics Laboratory, Faculty of Science and Engineering, Biochemistry, Åbo Akademi University, 20520 Turku, Finland; 3 Pharmaceutical Sciences Laboratory, Faculty of Science and Engineering, Pharmacy, Åbo Akademi University, 20520 Turku, Finland; 4 Department of Applied Physics, School of Science, Aalto University, Espoo, Finland

**Keywords:** *E. faecalis*, HPrK/P, HPr, Molecular docking, Molecular dynamics simulation, Structure-based drug design, Virtual screening

## Abstract

*Enterococcus faecalis*, a gram-positive bacterium, is among the most common nosocomial pathogens due to its limited susceptibility to antibiotics and its reservoir of the genes coding for virulence factors. Bacterial enzymes such as kinases and phosphorylases play important roles in diverse functions of a bacterial cell and, thus, are potential antibacterial drug targets. In Gram-positive bacteria, HPr Kinase/Phosphorylase (HPrK/P), a bifunctional enzyme is involved in the regulation of carbon catabolite repression by phosphorylating/dephosphorylating the histidine-containing phosphocarrier protein (HPr) at Ser46 residue. Deficiencies in HPrK/P function leads to severe defects in bacterial growth. This study aimed at identifying novel inhibitors of *E. faecalis* HPrK/P from a commercial compound library using structure-based virtual screening. The hit molecules were purchased and their effect on enzyme activity and growth of resistant *E. faecalis* was evaluated in vitro. Furthermore, docking and molecular dynamics simulations were performed to study the interactions of the hit compounds with HPrK/P. Among the identified hit molecules, two compounds inhibited the phosphorylation of HPr as well as significantly reduced the growth of resistant *E. faecalis *in vitro. These identified potential HPrK/P inhibitors open new research avenues towards the development of novel antimicrobials against resistant Gram-positive bacteria.

**Supplementary Information:**

The online version contains supplementary material available at 10.1007/s10822-022-00461-6.

## Introduction

Antimicrobial drugs have been used for decades to treat bacterial infections. However, many pathogenic bacteria fail to respond to treatment since these microorganisms have developed strategies to resist the effect of antibiotics [1]. Gram-positive bacteria are involved in more than half of the hospital-acquired infections and *Enterococci* are among the major pathogens to cause nosocomial bloodstream infections with high morbidity and mortality rate [2, 3]. Approximately, 80–90% of enterococcal infections are caused by *Enterococcus faecalis*, a commensal bacterium inhabiting the gastrointestinal tracts of humans [1, 4]. *E. faecalis* has developed resistance to commonly used antibiotics such as vancomycin, aminoglycosides, daptomycin, tetracycline, linezolid, and quinolones [5, 6]. In concert with antibiotic resistance, *E. faecalis* has also been reported to possess a reservoir of virulence genes and it can transfer these genes to other pathogenic bacteria [7]. Over the past few decades, *E. faecalis* has emerged as a significant pathogen due to its ability to cause a variety of infections such as urinary tract infection, bacteraemia, sepsis, pelvic infection, abdominal infection and rarely meningitis [8]. In *E. faecalis*, various mechanisms have been reported to counter the effect of antibiotics such as cell wall and cell membrane modifications, overexpression of efflux pumps, inactivation of antibiotics, and alteration of the drug targets [9]. Now, there is a need to identify new drug targets to reduce the risk of antibiotic resistance development. For instance, bacterial kinases and phosphorylases have been so far relatively overlooked in antimicrobial drug discovery. However, they play an important role in cell growth, intracellular metabolism, gene transcription, translation, signal transduction, and other essential cellular functions [10, 11] and, thus, could serve as suitable drug targets for the discovery of novel antimicrobials.

**Fig. 1:**
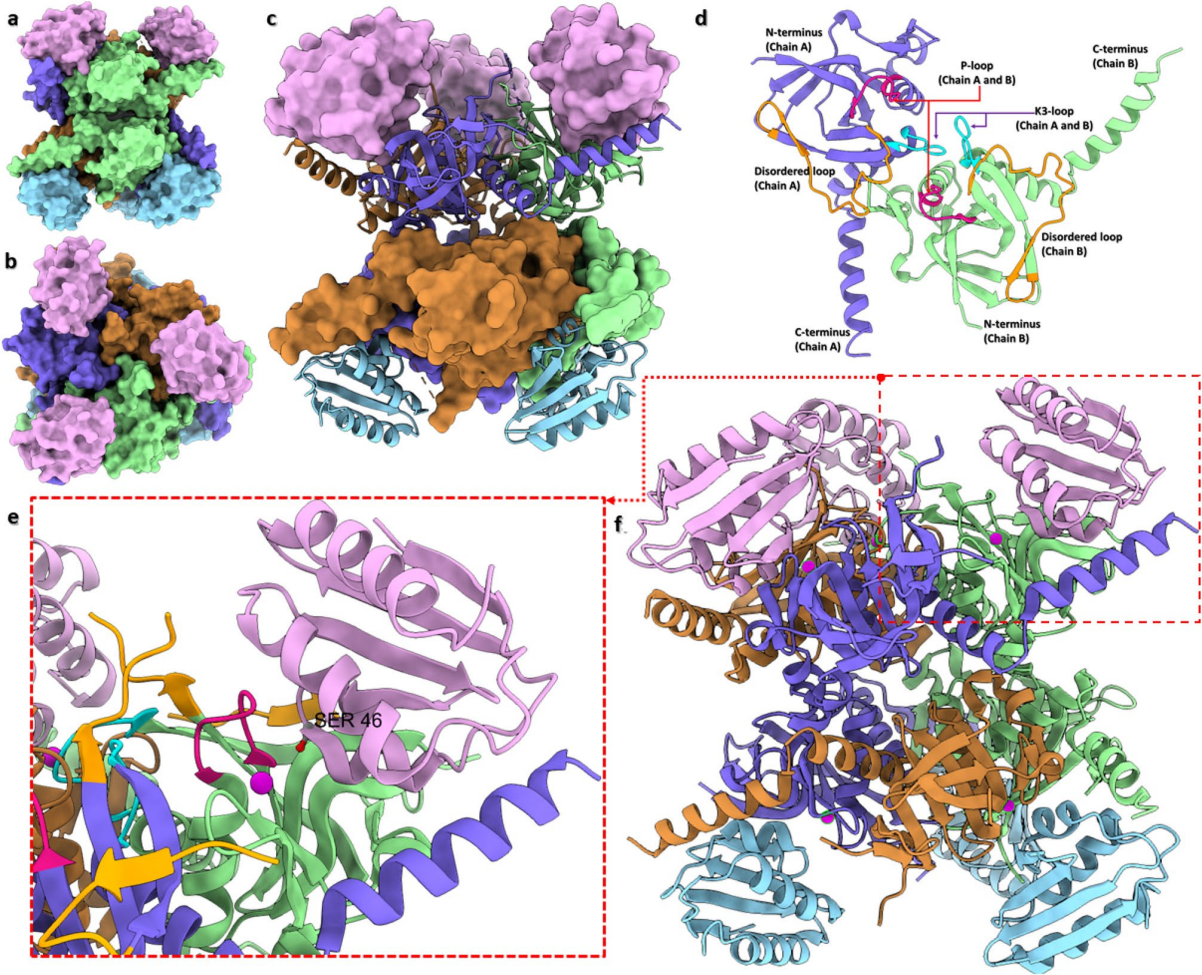
Crystal structure of the hexameric *L. casei* HprK/P in complex with *B. subtilis* HPr (PDB ID: 1KKL; [16]); **a** side view, surface representation; **b** top view, surface representation; **c** side view, mixed surface and cartoon representation. Color code: HPrK/P chains in violet blue, brown and light green; HPr protein in pink and light blue (top and bottom respectively). **d** Cartoon representation of the comparative (homology) model of the homodimeric *E. faecalis* HPrK/P (without ions). **e** Cartoon representation of the zoom-in view of HPr binding between two HPrK/P chains (the same structural assembly as in **a**–**c** and **f** panels). Serine 46 of HPr (in elemental ball-and-stick representation and labelled) is projected towards the co-crystallized calcium ion (magenta sphere) and P-loop of HPrK/P (dark pink); K3-loop is in cyan, and the disordered loop in orange. **f** The same side view as in **c** panel in cartoon representation

Bacteria utilize a preferred carbon source from the mixture of different sources through the process of carbon catabolite repression (CCR) that represses the expression of genes involved in the metabolism of secondary carbon sources [12]. CCR is regulated by a bi-functional enzyme that has kinase as well as phosphorylase activity, i.e., HPr Kinase/Phosphorylase (HPrK/P; EC:2.7.11.-, EC:2.7.4.-) [13]. The ATP-dependent HPrK/P phosphorylates Ser46 residue of histidine-containing phosphocarrier protein (HPr) of the phosphotransferase system (PTS) that plays an important role in sugar transport. In addition to kinase activity, it also carries the phosphorylase activity at the same active site. HPrK/P protein is a Ser/Thr protein kinase that shows no sequence similarity to eukaryotic Ser/Thr protein kinases [14–16]. The N-terminal (residues 1–134) of HPrK/P has no defined activity [14]. However, the catalytically active C-terminal domain (residues 135–319) contains a characteristic Walker motif A(G/A)xxxxGK(S/T) that forms the phosphate-binding loop (P-loop, residues 155–162), which binds the phosphate moiety of an incoming ATP nucleotide [17]. Residues 203–212 are important for the catalytic mechanism of phosphorylation/dephosphorylation and residues 266–271 (‘K3-loop’ that is connecting βK to α3) for dephosphorylation (UniProtKB; entry O07664, HPRK_ENTFA) [15]. Other important individual residues that have been identified forming the active/catalytic site are His140, Lys161, Asp179 (proton acceptor/donor), Arg245 in the flexible/disordered loop (residues 235–251), and the metal (Mg^2+^) binding residues Ser162 and Glu204. Crystallographic studies have revealed that HPrK/P is a hexameric enzyme that binds six HPr substrate molecules (Fig. 1a–c) [16]. The substrate-binding area of HPrK/P is exposed when the enzyme adopts an open conformation (~ 1400–2000 Å surface area) and six hydrogen bonds per HPr molecule stabilize the protein–protein interactions. Three molecules of HPr bind on the top and three HPr protein molecules bind on the bottom of the hexameric HPrK/P assembly. Two adjacent HPrK/P enzyme monomers interact with one HPr molecule; one HPrK/P monomer interacts with HPr via its catalytic site and the other monomer through its C-terminal helix [16] (Fig. 1d–f). The most contacts to HPr from one HPrK/P chain are initiated by βA (residues 135–140), the P-loop (connects βC to helix α1) and the βD–βE hairpin (residues 174–185). A conserved residue from the other subunit’s flexible/disordered loop, Arg245 is also important for stabilization of the interaction.

Previous studies have shown that a defect in kinase or phosphorylase activity of HPrK/P severely affects the carbon metabolism, growth, morphology and other cellular functions in *Staphylococcus xylosus, Lactobacillus casei, Mycoplasma pneumoniae and Bacillus subtilis* [17–20]. Therefore, bacterial kinases are also gaining pronounced attention as potential targets for antimicrobial drug discovery [11, 21, 22]. In our previous study, we observed an enhanced expression of *hprK* along with HPr-Ser46 phosphorylation in antimicrobial resistant *E. faecalis* strains [23]. This suggests that HPrK/P plays an important role in the fitness of antibiotic resistant *E. faecalis*. Therefore, the present study is aimed at identifying active-site targeted HPrK/P inhibitors against nisin-resistant *E. faecalis* that showed resistance to antibiotics [5]. Nisin is a class I bacteriocin that is used as a preservative for many food products. Here, we demonstrate that inhibition of bacterial HPr binding to HPrK/P is a strategy that limits the growth of resistant bacteria. To the best of our knowledge, this is the first study reporting HPrK/P inhibitors that have been identified using virtual screening and whose interactions with HPrK/P have been investigated with molecular dynamics (MD) simulations. The results of our study may facilitate the development of a new class of antimicrobials and thus opens up the possibility of combating multi-drug resistant Gram-positive bacteria such as *E. faecalis*.

## Materials and methods

### Materials for in vitro assays

Media components, Beef Extract (catalog number: RM002), Peptone (catalog number: CR001), Yeast Extract (catalog number: RM027) and Sodium Chloride (catalog number: TC046) for bacterial culture were purchased from HiMedia Laboratories (Mumbai, India). Anti-phosphoserine antibody was purchased from Sigma-Aldrich Chemical Co., St. Louis, MO (catalog number: SAB5200086-400UL). Inhibitor molecules were purchased from Enamine Ltd., Ukraine. PAGE reagents and buffers were purchased from Biorad and PVDF transfer membrane, 0.2 µm (Catalog number: 88520) was purchased from Thermo Fisher Scientific, Inc. Mini-PROTEAN® Tetra Cell, 2-gel, 10-well combs, 1.0 mm (catalog no. 1658003) from Biorad was used for PAGE and blotting was performed using semi-dry blotting unit (Scie-Plas Ltd., UK). Sensitive and resistant *E. faecalis* (Nis^R^-147) bacteria were previously isolated from raw buffalo milk in our lab [5, 23]. The nisin-resistant strain used here was found to be resistant to chloramphenicol, ampicillin, ciprofloxacin, rifampicin, vancomycin, carbenicillin, linezolid, oxacillin, and fosfomycin with minimum inhibitory concentrations (MICs) varying between 4 µg/mL (ciprofloxacin) and 512 µg/mL (fosfomycin) [5].

### Protein modelling

The amino acid sequence of *E. faecalis* HPrK/P protein (Accession Number: WP_002357309.1) was retrieved from the NCBI Protein database (https://www.ncbi.nlm.nih.gov/protein/). To find suitable template structures for comparative (homology) modelling, we used the Protein BLAST tool with default parameters against the Protein Data Bank (PDB) [24, 25]. The template crystal structures were then selected from the BLAST output based on the criteria of maximum identity percentage, a high BLAST score and a low E-value (PDB ID 1KO7: Query coverage = 94%, Percent identity = 51.70%, E value = 10^–105^; PDB ID 1KKL: Query coverage = 58%, Percent identity = 71.04%, E value = 2 × 10^–89^). The Expect value (E) describes the number of "expected" hits of similarity in a database. It decreases exponentially as the score (S) of the match increases. The crystal structures of the full-length HPrK/P protein of *S. xylosus* (PDB ID: 1KO7; resolution 1.95 Å) [26] and the C-terminal catalytic domain of *L. casei* HPrK/P (PDB ID: 1KKM; resolution 2.80 Å, related to 1KKL but containing also the coordinates for the flexible/disordered loop) [16] were retrieved from the PDB and further used to prepare a full-length monomeric model and a homodimeric C-terminal catalytic domain model, respectively. The initial monomeric model based on the single template (1KO7, A chain) was built with the Swiss-Model server (https://swissmodel.expasy.org) [27]. As the template structure lacks the coordinates for the long flexible/disordered loop, the missing region was modelled in by the server. This model was utilized in the virtual screening phase. For the binding mode studies, we built another full-length monomeric model utilizing multiple HPrK/P template structures, some of which contained coordinates also for the disordered loop as well as the complete C-terminus. The following template structures were selected from the BLAST results: A chains of PDB IDs 1KNX [28] (from *M. pneumoniae*, resolution 2.50 Å), 2QMH [29] (from *L. casei*, resolution 2.60 Å), 1KO7, 1KKL [16] (from *L. casei*, resolution 2.80 Å) and 1JB1 [15] (from *L. casei*, resolution 2.80 Å). Apart from the Swiss-Model-generated monomeric model, the modelling alignments were prepared with the alignment tools (align2d, salign) of Modeller 9.20 [30]. Ten comparative models were built using Modeller 9.20 for both the monomeric and the dimeric forms. The model having the lowest discrete optimized protein energy (DOPE) score was selected for the monomeric form whereas the model with the lowest possible DOPE score and the best stereochemical quality was selected for the dimeric structure [31]. For the molecular dynamics (MD) simulations, two alternative homodimeric models were used: one without ions (Fig. 1d) and the other with an added Mg^2+^ and a phosphate ion to observe the possible effect of metal coordination on inhibitor binding. The phosphate coordinates were obtained from PDB ID: 1KO7 and Mg^2+^ coordinates from PDB ID: 1KKL (Mg^2+^ was replaced for the original Ca^2+^ since HPrK/P uses Mg^2+^ for the catalysis). The characteristic P-loop (Fig. 1d, e) offers the binding site for the phosphate ion (normally occupied by the β-phosphate of the bound nucleotide), [26] while Mg^2+^ stays close to the phosphate ion and facilitates the phosphorylation of Ser46 at HPr. No constraints were needed to keep the ions in their positions during the simulations. The quality assessment of the models was performed using Procheck [32], ProSA [33, 34], and QMEANDisCo scores [35].

### Virtual library screening

Virtual screening was performed with the INVENTUS Drug Discovery suite v1.1 (Novo Informatics Pvt Ltd). First, the active site was predicted on the Swiss-Model-generated monomeric HPrK/P enzyme model using the PocketDetector™ module of INVENTUS as per the automated active site detection, docking, and scoring (AADS) protocol [36]. The selected active site was formed by the P-loop residues as well as other residues important for the catalytic mechanism of phosphorylation and/or dephosphorylation; residues 158–163, 166–167, 170–171, 206, 232, 267–275, 277. The coordinates of this site were then uploaded to the HitsGen™ module (RASPD protocol) [37] which was used for the virtual high-throughput screening of an inbuilt INVENTUS library that has around four million compounds. HitsGen™ employs a fast-quantitative structure–activity relationship (QSAR)-based methodology to screen for compounds whose physico-chemical properties are complementary to the properties of the target binding site, without actually having to dock the molecules to the target. The ligand binding energy (bio-affinity score) of the screened compounds is estimated with the robust inbuilt QSAR equation that was developed using a representative training set of experimental drug-receptor complexes [37]. Therefore, apart from the structural properties of the given binding site, the following ligand property criteria were used to screen through the compound library: hydrogen bond donors = 0–5, hydrogen bond acceptors = 0–10, LogP (SlogP) = 0–5, molar refractivity = 40–130, number of aromatic atoms = 15–57, length of molecule = 10–100 Å, total number of atoms = 10–100, and binding energy < − 5 kcal/mol. These criteria were set based on a previously described, potent (IC_50_ 17–18 µM at pH 7.0–8.0, respectively), ditopic 2-aminobenzimidazole-type inhibitor “3-B-3” of *B. subtilis* HPrK/P [10] (Table 1). Three inhibitor candidates from overall 50 hits were selected for in-vitro activity evaluation based on the best (most negative) bio-affinity score.

**Table 1:** Reference inhibitor and top-3 hit compounds from virtual screening using HitsGen™ of INVENTUS v. 1.1

Compound ID	IUPAC Name and 2D structure^a^	BioAff^b^ (kcal/mol)	Donor	Acceptor	LogP	Molar Refractivity (m^3^/mol)	MW (g/mol)
Benzimidazole inhibitor 3-B-3^c^	2-(3-benzyl-2-imino-2,3-dihydro-1*H*-benzo[*d*]imidazol-1-yl)-*N*-((*E*)-4-((*E*)-(2-(2-(2-imino-3-phenyl-2,3-dihydro-1*H*-benzo[*d*]imidazol-1-yl)acetyl)hydrazineylidene)methyl)benzylidene)acetohydrazide	–	4	12	6.35	200.78	688.78
NITSKI152	(*E*)-3-(3-hydroxy-4-methoxyphenyl)-1-(6-methoxynaphthalen-2-yl)prop-2-en-1-one	− 5.09	1	4	4.46	98.76	334.4
NITSKI8583	5-bromo-1-[2-(2,5-dimethoxyphenyl)-2-oxoethyl]pyridin-2-one	− 5.08	0	4	2.7	97.45	352.18
NITSKI5508	*N*-[[4-(diethylsulfamoyl)phenyl]methyl]-1*H*-pyrrole-2-carboxamide	− 5.02	2	4	4.15	100.7	335.4

^a^2D structures were sketched using ChemDraw version 21.0.0.28 (PerkinElmer Informatics, Inc 1998–2022)

^b^Bio-affinity score of HitsGen™ is predicted with a generic QSAR-type equation based on structural and physico-chemical properties of both the screened ligands and the target binding site

^c^Ref. [10]

### In-vitro evaluation

#### Effect of inhibitors on growth and morphology of resistant *E. faecalis*

To evaluate the effect of the three identified candidate inhibitors on bacterial growth, Colony-Forming Units (CFU) were counted. Resistant *E. faecalis* was grown in the presence of 1 mM inhibitors (100 mM stock dissolved in DMSO) and without any inhibitor as a control from 0 to 8 h and plated on nutrient agar Petri dish. Then, CFU mL^−1^ was calculated. To visualize the effects of inhibitors on bacterial morphology, Gram staining of the bacteria was done and examined under a microscope (×1000).

#### Detection of HPr-Ser-phosphorylation using western blotting

To determine the effect of the candidate inhibitors on the activity of HPrK/P, western blotting was performed. Nisin resistant *E. faecalis* strain was grown in nutrient broth till the exponential phase in the presence of 1 mM inhibitors and without any inhibitor as a control, harvested and washed with ice-cold 10 mM Tris–HCl buffer. Cells were lysed using bead beater (FastPrep®-24, Bio101/Savant, Farmingdale, NY), followed by centrifugation at 10,000 rpm at 4 °C for 20 min. The crude sample was loaded on 12% SDS-PAGE without heating. Anti-phosphoserine antibody was used to detect the serine phosphorylation of the HPr protein as described in [23]. The experiment was performed in triplicate and relative density was analysed using ImageJ (1.51v 9). Statistical analysis was performed using GraphPad Prism 8.0.0.

### Computational binding mode analysis of the hit compounds

#### Molecular docking

Since our virtual screening method utilized only the properties of the reference ligand and the selected pocket in a monomeric subunit, we did not get any realistic view of the possible binding modes of the ligands at the hexameric enzyme. Thus, the binding mode of the three candidate compounds at HPrK/P was investigated by molecular docking using the Glide docking tool [38, 39] of Schrödinger’s Maestro Molecular Modeling Suite (Schrödinger Release 2022-1: Maestro, Schrödinger, LLC, New York, NY). The protein structures were also visualised using the Pymol Molecular Graphics System (version 2.3, Schrödinger, LLC). The structures of the reference and hit compounds were prepared with the LigPrep tool of Maestro. The missing hydrogen atoms were added, and alternative protonation/ionization states (if any) were generated at pH 7 ± 2 using Epik [40]. Finally, the structures were optimised using the OPLS4 force field [41] to get low-energy 3D conformers of the ligands. The reference and hit compounds’ binding was investigated at all the three Modeller-built comparative models of HPrK/P (i.e., the multi-template-based monomeric model and the dimeric model with and without the Mg^2+^ and phosphate ions). All three models were processed with the Protein Preparation Wizard [42] of Maestro: the missing hydrogen atoms were added, and the hydrogen bond network was optimized with PROPKA at pH 7.0 [43, 44]. The protein models were energy-minimized using the OPLS3e force field [41] with the convergence criteria of the root-mean-square deviation (RMSD) 0.3 Å for all heavy atoms. Due to the absence of a co-crystallized ligand, we identified the docking sites using Maestro’s SiteMap tool [45, 46], which also detected the same binding pocket whose properties had been used for the virtual screening protocol. However, that site was not the best-ranked among the pockets analysed. Moreover, in the virtual screening phase we had not considered the multimeric form of the enzyme and observed that the initial site detected in the monomeric model would not even exist in the hexameric molecule as the K3-loop of an adjacent subunit would be located at that site (thus, we named the site the ‘K3-loop site’). The best-ranked pocket by SiteMap was the substrate-binding pocket on the phosphate-binding side of the P-loop, next to the flexible/disordered loop. Since the reference inhibitor has also been suggested to act on the HPrK/P substrate site [10], we chose both the initial K3-loop site in the monomeric model and the substrate binding site in the dimeric models for the binding mode studies. With Maestro’s Receptor Grid Generation tool, the docking site for the monomer model was defined using the same residues as at the virtual screening stage. The size of the inner grid box was kept at the default size (10 × 10 × 10 Å^3^) whereas the outer box size was set to 30 × 30 × 30 Å^3^. For the dimeric models (with and without ions), the centre of the docking grid was defined by the residues forming the substrate-binding site (Arg245, Thr294, Leu301, Ile305 and His140′, Ser157′, Asp179′, Arg180′, Ile198′, Leu199′, Glu204′; a prime (′) denotes the residues from the other subunit). The inner and outer box sizes were set to 15 × 15 × 15 Å^3^ and 35 × 35 × 35 Å^3^, respectively. The most suitable grid box size in each case was selected based on the test docking results of the reference inhibitor. The maximum length of the ligands to be docked was set to 20 Å for all three models. The docking was carried out using the Glide extra precision (XP) [47] mode with flexible ligand sampling. The Epik [40] state penalties for different ionization states/tautomers of the compounds were added to the final docking scores. This can be crucial when ranking compounds with more than one ionization state/tautomer. For each ligand, ten poses were taken for post-docking minimization and a minimum of one pose was generated per ligand.

#### Molecular dynamics simulations

Molecular dynamics (MD) simulations of the docked ligand-enzyme complexes were carried out for 100 ns with Desmond (Schrödinger Release 2022-1: Desmond Molecular Dynamics System, D. E. Shaw Research, New York, NY, 2022. Maestro-Desmond Interoperability Tools, Schrödinger, New York, NY, 2022) [48] using the OPLS4 force field [49]. Three replicate simulations for each system were performed. The simulation systems were prepared with the System Builder tool of Desmond. An orthorhombic simulation box with Periodic Boundary Conditions (PBC) was filled with single point charge (SPC) water [50] and a 10-Å buffer space was left between the solute (ligand-enzyme complex) and the box edge. In addition, the system was neutralized using an appropriate number of chloride ions for each model. The simulation system was initially relaxed with and without restraints on the solute heavy atoms using the following stepwise relaxation protocol: 12 ps of Brownian dynamics in NVT ensemble at 10 K using a Berendsen thermostat and a 1-fs time step with restraints; 12 ps of Langevin dynamics in NPT ensemble at 300 K and 1 atm pressure using a Berendsen thermostat and barostat with restraints and 24 ps without restrains. The actual production simulations were performed in NPT ensemble for 100 ns with a 2-fs time step. The temperature was set at 300 K with the Nosé-Hoover chain thermostat [51, 52] and pressure at 1.01325 bar with the Martyna-Tobias-Klein barostat [53] using isotropic coupling and relaxation time of 1 ps and 2 ps, respectively. For handling the short-range Coulombic interactions a cut-off radius of 9.0 Å was used whereas the *u*-series decomposition of the Coulomb potential was used for the long-range electrostatic interactions [54]. The simulation trajectories were analysed using the Maestro in-built Simulation Interactions Diagram tool after which the interaction analysis data generated was processed with Microsoft Excel360 to prepare illustrating graphs to aid the analysis.

#### Binding free energy analysis

Molecular Mechanics-Generalized Born Surface Area (MM-GBSA) method implemented in Prime [55, 56] (Schrödinger Release 2022-1: Prime, Schrödinger, LLC, New York, NY, 2022) of Maestro was used to predict the binding free energy (ΔG_bind_) of the docked ligands at the HPrK/P substrate binding site. The ΔG_bind_ was calculated using the following equation:

$$\Delta {\text{G}}_{{{\text{bind}}}} = {\text{ G}}_{{{\text{complex}}}} - \, \left( {{\text{G}}_{{{\text{receptor}}}} + {\text{ G}}_{{{\text{ligand}}}} } \right)$$

where, G_complex_ is the free energy of the ligand–protein complex, G_receptor_ is the free energy of the protein and G_ligand_ is the free energy of the ligand. These energy values are calculated as

$$G = \, G_{MM} \, + \, G_{solv}$$

where, *G*_*MM*_ is the calculated molecular mechanics (MM) energy for the force field applied, and *G*_*Solv*_ is the solvation energy in the generalized Born approximation. Prime/MM-GBSA analysis was carried for both the initial docking poses and the final simulated poses after MD using the ‘thermal_mmgbsa.py’ script from Schrödinger with the variable-dielectric generalized Born model (VSGB 2.1 solvation model) [57] and the OPLS4 force field [49]. The Prime/MM-GBSA energies were calculated for the complete 100-ns trajectories by evaluating the snapshot structures of the complexes at every 100 ps. The results from the replicate simulations were averaged.

## Results and discussion

### Structural analysis and quality of the HPrK/P comparative models

**Fig. 2:**
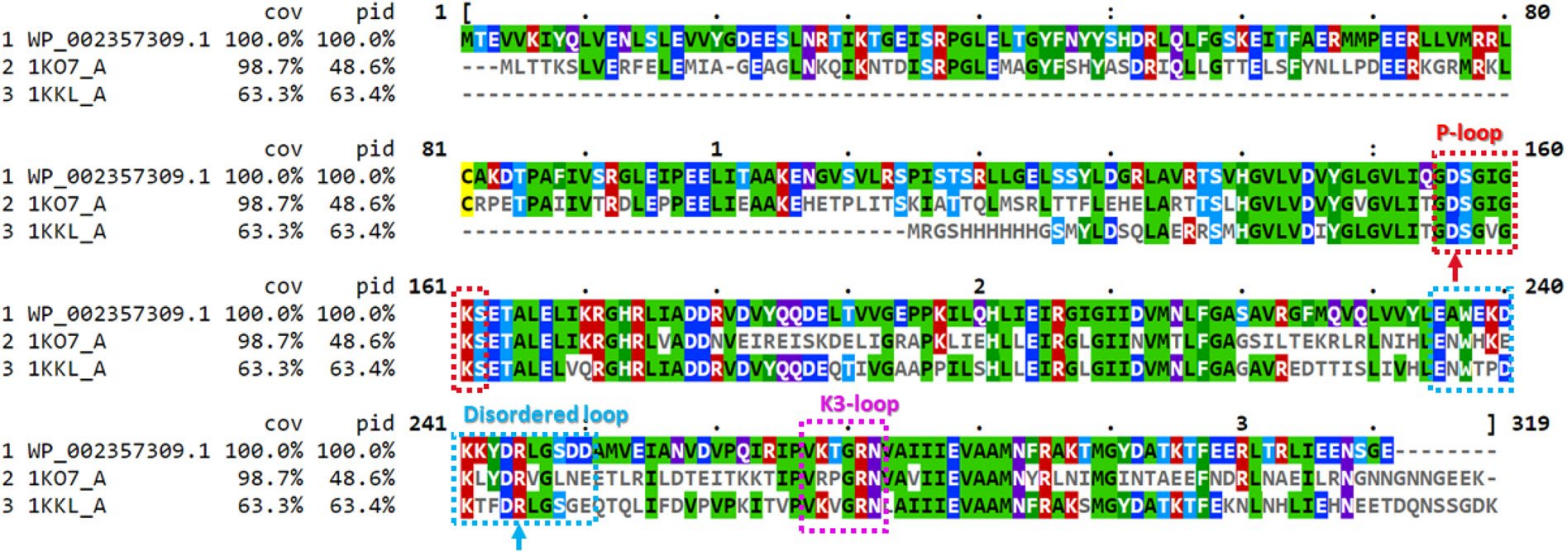
Multiple sequence alignment of *E. faecalis* HPr kinase/phosphorylase (HPrK/P) sequence (WP_002357309.1/UniProtKB entry: O07664) with the Protein Data Bank-retrieved sequences of selected template crystal structures: both the structure of *S. xylosus* HPrK/P (PDB ID: 1KO7, A chain) and the structure of *L. casei* HPrK/P (PDB ID: 1KKL, A chain) were among the templates used for the full-length monomeric model. The three important loops; P-loop (residues 155–162), flexible/disordered loop (residues 235–251) and K3-loop (residues 267–272) are highlighted with red, cyan and pink boxes, respectively. A red and a cyan color arrow point to the catalytic Asp-156 and Arg-245 residues at the P-loop and the disordered loop, respectively. The multiple sequence alignment was created with the Clustal Omega tool [58] and visualized with Mview [59] at EMBL-EBI [60]; cov—sequence coverage; pid—percent identity; identical amino acids are colored according to their physicochemical properties. The coordinates for the disordered loop residues W233-L245 and the C-terminal residues 299–314 are missing from the *S. xylosus* HPrK/P crystal structure (1KO7, A chain), whereas the N-terminal residues up till E135, the disordered loop residues K241-G249, and the C-terminal residues T311-K319 are missing from the *L. casei* structure (1KKL, A chain)

To illustrate the sequence identities and locate the key C-terminal domain loops and some of the active site residues, a multiple sequence alignment of the *E. faecalis* HPrK/P sequence with the PDB-retrieved sequences of the *S. xylosus* and *L. casei* HPrK/P crystal structures is shown in Fig. 2.

The Modeller modelling alignments for the monomeric and dimeric models are shown in Supplementary Information (p. S2, S3). The best comparative models of *E. faecalis* HPrK/P of each type (monomer/dimer) were selected based on the DOPE score (− 32,637.54/− 39,614.91 for the monomeric/dimeric models, respectively) and the stereochemical quality of the models. The stereochemical quality of the generated models was assessed using the Ramachandran plots generated by PROCHECK [32]. The Ramachandran plot of the monomeric model showed 92.0% of the residues in the most favoured regions, 6.9% in additionally allowed regions, 0.7% in generously allowed regions and only 0.4% (Asp179) in a disallowed region (see Supplementary Information, Fig. S1). On the other hand, the Ramachandran plot of the homodimeric protein model showed 94.2% of the residues in the most favoured regions, 5.1% in additionally allowed regions, 0.0% in generously allowed regions and only 0.6% (Asp179 in both chains; Asp46 and Asp224 according to the dimeric model’s residue numbering: 1st chain 1–178, 2nd chain 179–356) in disallowed regions (Fig. S1). The residue found in the disallowed region has been identified as the proton donor/acceptor in the dephosphorylation/phosphorylation reaction, respectively (UniProtKB entry O07664). It is located at the tip of the βD–βE hairpin loop and its phi/psi angles differ from the usual values in the template structures (e.g. 1KKM and 1KO7), explaining why it is in the disallowed region also in the models. The RMSD of Cα atoms of the full-length monomeric model was found to be 0.747 Å when superimposed on one of the templates (PDB ID: 1KO7, A chain) and the RMSD of the C-terminal catalytic domain models of the homodimeric HPrK/P was found to be 0.272 Å when superimposed on the template (PDB ID: 1KKM, A and B chains) using PyMol. The overall model quality and degree of nativeness for both the models were evaluated using the ProSA web server. The Z-score was found to be − 6.92 for the monomer model and − 5.67 for the homodimeric protein model, confirming that the model structures are within the Z-score range that is typically found for native proteins of similar size (Fig. S1). The Qualitative Model Energy Analysis (QMEAN) was done using the QMEANDisCo scoring function. It includes a term for estimating the local per-residue quality based on the similarity of pairwise residue-residue distances in the model compared to the sets of distance constraints obtained from homologous structures [35]. The Global Score is an average of the per-residue lDDT (the local Distance Difference Test) score that ranges from 0 to 1 (1 is good) and was found to be 0.73 ± 0.05 for the monomeric protein model and 0.78 ± 0.05 for the homodimeric protein model (more details are provided in Supplementary Information, Fig. S1). We also compared our models with the artificial intelligence-based AlphaFold [61] model of *E. faecalis* HPrK/P (alphafold.ebi.ac.uk). When aligned with the AlphaFold full-length monomeric model, the RMSD of Cα atoms for our monomeric models was 0.994 Å (initial model) and 0.852 Å (multi-template model) whereas the RMSD was only 0.601 Å for the C-terminal catalytic domains in our dimeric models. The disordered loop conformation and the C-terminal helix orientation in the dimeric models was very similar to the AlphaFold structure (more similar than in the monomer models). On the other hand, the AlphaFold model was very similar (RMSD of the Cα atoms: 0.585 Å) to the template that was used for building the dimeric model (PDB ID: 1KKM, A and B chains).

### Drug screening and docking

The virtual screening of putative HPrK/P inhibitors was performed with the HitsGen™ module of the INVENTUS software suite by (i) scanning the inbuilt compound library based on physicochemical and structural properties of a known HPrK/P inhibitor [10] and the selected binding site (K3-loop site) and (ii) ranking the hit molecules with the bio-affinity score. Three hit compounds with the best score were selected for further investigations (Table 1).

**Fig. 3:**
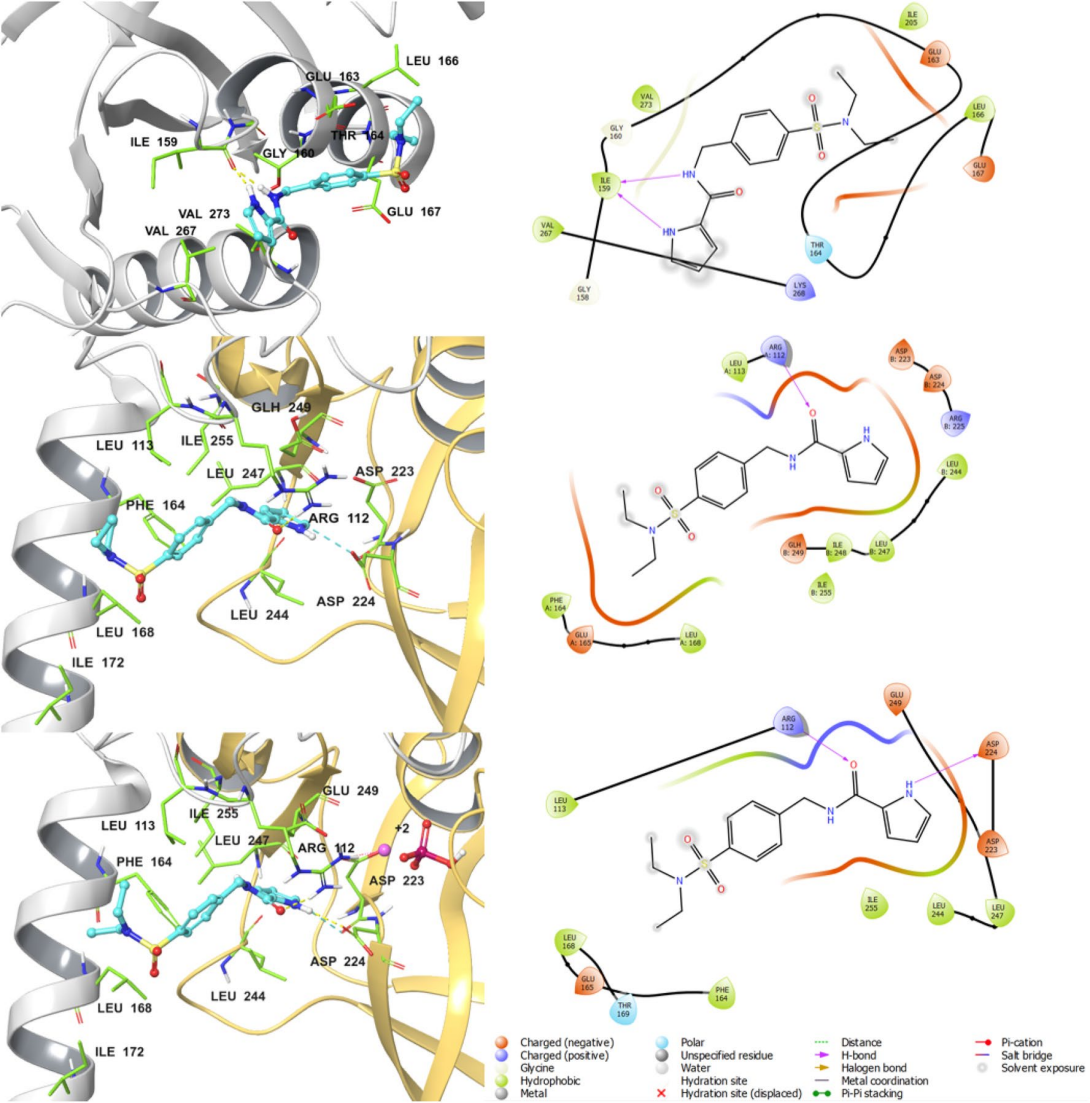
The virtual screening hit compound NITSKI5508 docked in the *E. faecalis* HPrK/P monomeric and substrate-binding site. The docking site interactions are shown at all the three HPrK/P models in 3D (left) and 2D (right). Top: full-length monomeric HPrK/P model; middle: homodimeric model of the HPrK/P C-terminal catalytic domain; bottom: homodimeric model of the HPrK/P C-terminal catalytic domain with Mg^2+^ and PO_4_^3−^ ions. Left panel: one monomer chain of HPrK/P is shown in gray color cartoon representation while the other one is in yellow. The docked ligand is shown in ball-and-stick representation (cyan carbon atoms) and the binding site residues are in sticks (green carbon atoms); Mg^2+^ ion is shown as a pink sphere and the phosphate ion in ball-and-stick representation; oxygen atoms are shown in red, nitrogen atoms in blue, phosphorus in dark pink, hydrogen atoms in white. Yellow dashed lines indicate hydrogen bond interactions. Residue numbering in the dimeric models: 1st monomer chain 1–178, 2nd monomer chain 179–356 (corresponding to residues 134–311 in the monomer model)

The binding mode of the hit compounds at HPrK/P was further investigated by docking them both to the K3-loop site in the monomeric model and the substrate-binding area of the dimeric HPrK/P models. The initial assessment of the docked poses revealed that the compounds had been successfully docked at these sites; in the K3-loop site between the P-loop (residues 155–162) and the K3-loop (residues 266–271) and in the substrate-binding area, close to the flexible loop region that contains the catalytically critical Arg245. The initial docking scores (Table 2) are seemingly poor for all three hit compounds at both sites, although slightly better at the substrate binding site, and there might be several reasons to this. Either the compounds do not bind to the particular site, or they bind with only low affinity, or the comparative models of HPrK/P are not accurate, especially regarding the conformation of the side chains and flexible regions. The binding site conformation crucially affects the ligand docking poses and possible ligand–protein interactions. Since the flexible/disordered loop containing Arg245 is part of the substrate binding site, the site is likely to be very dynamic and is affected by the binding partner’s size and other properties. Therefore, we further investigated the docked poses using molecular dynamics (MD) simulations to see whether the docked complexes were stable and if we could see any improvement in the estimated binding free energies (ΔG_bind_, kcal/mol) during the MD simulations (Table 2). Of note, the reference inhibitor 3-B-3 most likely binds to the substrate-binding site and its ditopic structure is beneficial for the inhibitory activity [10]. This is consistent with the predicted binding affinities that are much better for the substrate-binding site than for the K3-loop site. The docked pose of NITSKI5508 in all three HPrK/P models before the MD simulation is shown in Fig. 3 (the poses of the other hits are presented in Supplementary Information, Figs. S2, S3).

**Table 2:** Docking scores and calculated free energies (ΔG_bind_) of binding for the reference and hit compounds docked at the monomer and dimer models of the *E. faecalis* HPrK/P

Compound ID	Glide XP Score (kcal/mol)			ΔG_bind_ (kcal/mol) (before MD simulation/mean ± SD from the last 50 ns of MD replicated 3 times		
	Monomer K3-loop site	Homodimer HPr binding site	Homodimer with ions HPr binding site	Monomer K3-loop site	Homodimer HPr binding site	Homodimer with ions HPr binding site
Benzimidazole inhibitor 3-B-3^a^	− 3.94	− 6.26	− 6.91	− 41.99/− 48.21 ± 7.61	− 62.55/− 80.22 ± 6.92	− 62.85/− 72.09 ± 8.39
NITSKI152	− 1.64	− 3.53	− 5.08	− 36.62/− 45.10 ± 6.77	− 35.02/− 50.65 ± 4.85	− 25.092/− 42.80 ± 3.85
NITSKI8583	− 2.49	− 3.13	− 3.69	− 23.76/− 33.87 ± 5.02	− 36.65/− 45.64 ± 4.21	− 31.89/− 30.42 ± 6.23
NITSKI5508	− 2.45	− 2.92	− 4.20	− 37.48/− 35.58 ± 4.27	− 37.93/− 39.39 ± 5.02	− 33.09/− 36.57 ± 4.47

^a^Ref. [10]

### MD simulation analysis

#### Stability of the complexes

The stability of the ligand-HPrK/P (monomer and homodimer models with and without ions) interactions was assessed by observing the changes in the protein backbone RMSD and backbone root-mean-square fluctuation (RMSF) per residue as well as the ligand RMSD with respect to the protein during the MD simulation (Supplementary Information, Figs. S4–S10). In general, the protein RMSD converged for all models with all ligands. However, the RMSD values were significantly higher for the monomeric model. The RMSF plot reveals that it is mainly the N-terminal domain residues of the monomeric model and the C-terminus of all models that are fluctuating significantly, although the flexible/disordered loop at the substrate-binding site also shows a visible peak in the fluctuation graphs. Ligand fluctuations with respect to the protein revealed that all hit compounds found a relatively stable pose in the monomeric model towards the end of the simulation although the initial fluctuations were large especially for NITSKI152 and NITSKI5508. On the other hand, positions of NITSKI8583 and NITSKI5508 fluctuated more in the dimeric models than the position of NITSKI152 (Fig. S10).

#### Interaction analysis

Detailed interaction data from the MD simulations of the ligands with the HPrK/P models are presented in the Supplementary Material (Table S1, Figs. S11–S16). At the substrate-binding site, many ligand–protein interactions were observed with the highly disordered loop (residues 235–251) and its surrounding region. This region plays a significant role in the protein–protein interaction of HPrK/P and its substrate HPr [62]. Therefore, the binding of inhibitors in this region can interfere with the binding of HPr and, thus, will impede the catalytic reactions. The impairment will further abolish the carbon catabolism, required for the essential functioning of a bacterial cell [17]. For example, in the simulations of the dimeric models, Arg245 and Leu246 of the flexible loop frequently formed hydrogen bonding or hydrophobic interactions with the ligands, respectively. On the other hand, in the simulations of the monomeric model, especially residues Ile159 (P-loop region) and Val273 (K3-loop region) formed polar (via the backbone atoms) or hydrophobic interactions (via the side chains) with the ligands at the K3-loop site. Furthermore, it has been shown that residues from the C-terminal α-helix (α3) of HPrK/P also take part in binding the substrate [16]. In the simulations of the dimeric models, Phe297 and Leu301 of the C-terminal helix form aromatic or hydrophobic interactions with the ligands. In addition to the direct hydrogen bonding interactions, many water-mediated interactions also contribute to the binding affinities of the ligands (Figs. S11–S16).

##### Binding free energy calculations

During the MD simulations, the Prime/MM-GBSA binding free energies were calculated for all the three hit compounds at the three different HPrK/P protein models at 100-ps intervals (Table 2 and Supplementary Information, Fig. S17). In general, the binding free energies of the hit compounds improved somewhat during the MD simulations as they found a more favorable pose compared to the initial docking pose. The reference compound 3-B-3 shows the best binding free energies throughout the simulations at both sites, but the energies are much better at the substrate binding site. On the other hand, NITSKI152 exhibits the best binding free energies of the hit compounds at both sites, and comparable energies at the K3-loop site with the reference compound. The presence of the added ions in the dimer model had a positive effect on the Glide XP docking scores especially for the hit compounds, but this effect was not seen in the Prime/MM-GBSA energies before or during the MD simulations (Table 2). The effect of the pose refinement by MD simulations was evidently beneficial for all the other compounds but not NITSKI5508. However, the improvement in the average binding energy was not significant for the reference compound at the K3-loop site, which would support the fact that it rather binds at the substrate-binding site. Despite the seemingly unfavorable binding energies, a crude decoy docking test suggests that it was not mere serendipity that the three hit compounds were discovered using the K3-loop site (Supplementary Information p. S19, Fig. S18). In case the enzyme is an obligate hexamer to be functional, these compounds may disrupt the multimerization process once the protein chains have folded and are assembled into the hexameric form. For example, human thymidylate synthase (EC 2.1.1.45) is an obligate dimer and indeed, it has been shown experimentally that the dimeric enzyme can be disrupted by small molecules, shifting the equilibrium to monomers that are then degraded easily [63].

### Effect of inhibitors on morphology and growth of resistant strain

**Fig. 4:**
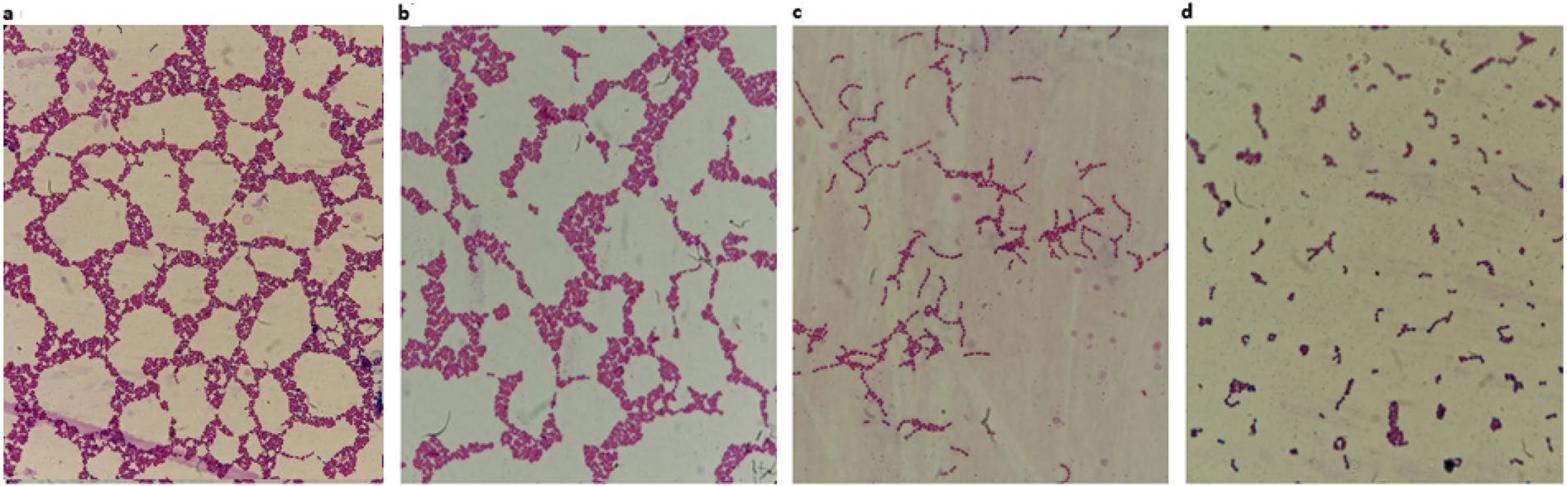
Gram staining of *E. faecalis* examined under a light microscope (magnification ×1000). Resistant *E. faecalis* strain-control (**a**), effect of NITSKI152 (**b**), NITSKI8583 (**c**), and NITSKI5508 (**d**) on resistant *E. faecalis*

**Fig. 5:**
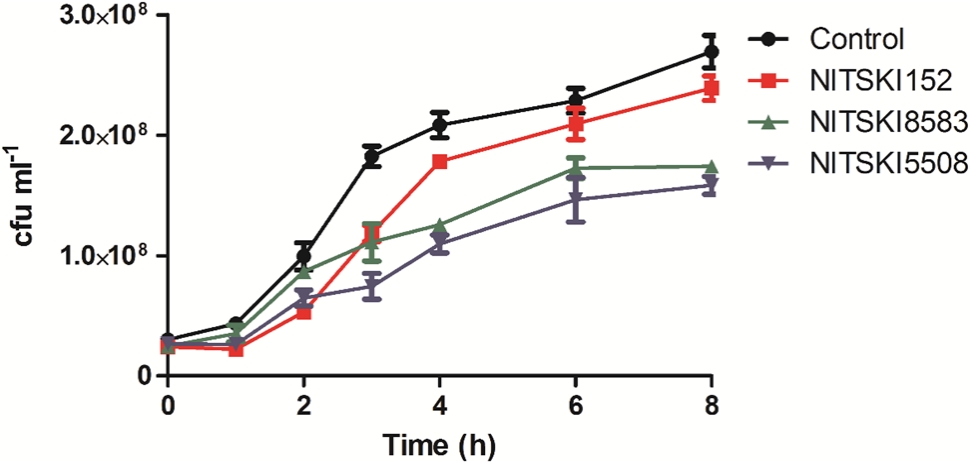
Growth curve of resistant *E. faecalis*. Resistant strain grown in the absence (control) and in the presence of NITSKI152, NITSKI8583 and NITSKI5508 compounds. Data points are presented as mean (n = 3) ± SD

Microscopy results showed that resistant *E. faecalis* formed clumps and a web-like structure by joining the chains (Fig. 4a). However, in the presence of inhibitors, NITSKI8583 and NITSKI5508, the clump formation and webbing were observed to be significantly reduced (Fig. 4c, d). The growth of the resistant strain was also found to be significantly decreased when grown in the presence of these inhibitors (Fig. 5). These results well corroborate with the previous reports that showed that deficiency of HPrK/P protein leads to a pleiotropic effect on bacterial physiology [64] and inactivation of HPrK/P activities results in the deleterious effect on the bacterial growth [20]. HPrK/P deficient strains are unable to grow on the phosphoenolpyruvate:glycose phosphotransferase system (PTS) and on most non-PTS carbohydrates [65].

### Western blot analysis of HPr-Ser-phosphorylation

**Fig. 6:**
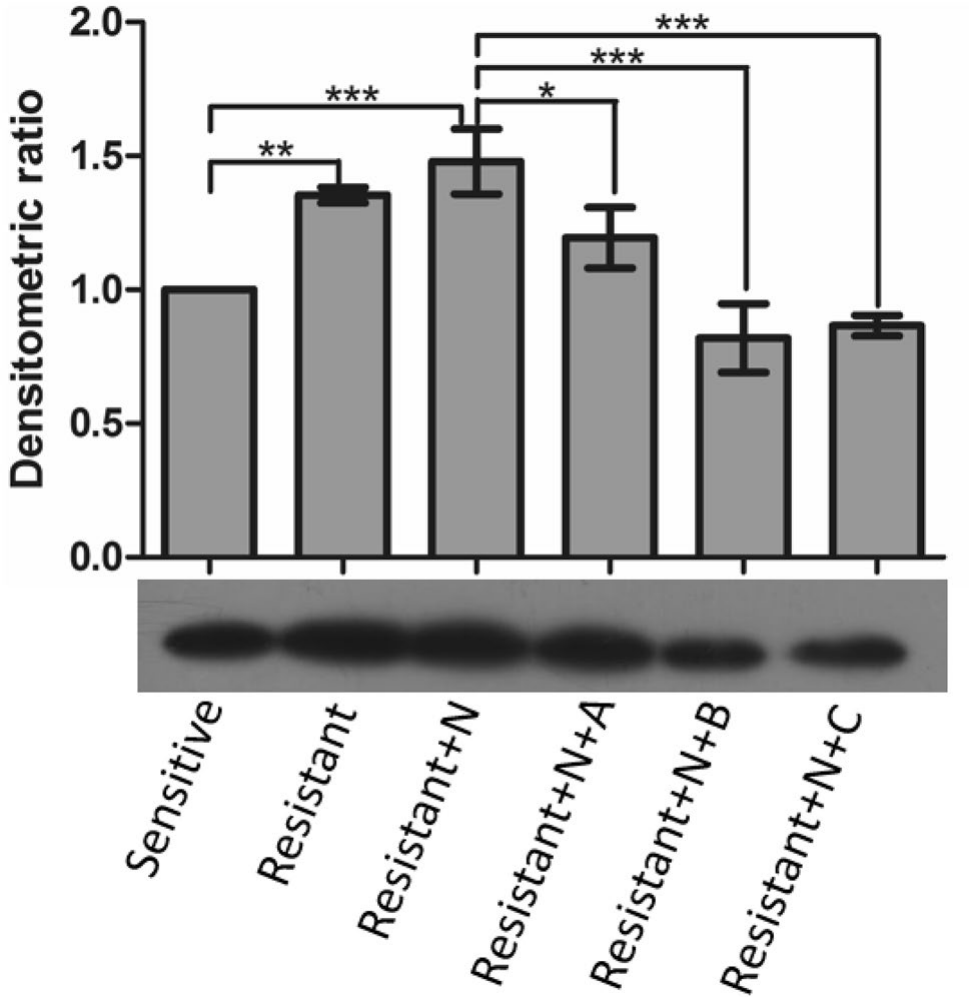
Western blot analysis of HPr-Ser phosphorylation of *E. faecalis*: HPr-Ser46 phosphorylation in a sensitive and a resistant strain. Sensitive: nisin sensitive *E. faecalis*; Resistant: nisin resistant *E. faecalis*; Resistant + N: nisin resistant *E. faecalis* grown in the presence of nisin; N: Nisin; A: NITSKI152; B: NITSKI8583; C: NITSKI5508. Data represent relative density mean (n = 3) ± SD. Statistical analysis was performed using One Way ANOVA Tukey test. *P < 0.05, **P < 0.01and **P < 0.001

When resistant *E. faecalis* was grown in the presence of inhibitors, the phosphorylation of HPr-Ser46 residue was found to be decreased significantly. Inhibitors NITSKI8583 and NITSKI5508 were found to be the most potent to reduce the activity of HPrK/P, which was in concordance with the decreased HPr-Ser phosphorylation (Fig. 6). In our previous study, we found that with the progression of nisin resistance in *E. faecalis*, the phosphorylation of the Ser46 residue of HPr protein was enhanced [23]. Researchers [64] also reported that inactivation of *hprK* gene results in the loss of kinase and phosphorylase activity, which severely reduced the growth of bacteria.

## Conclusions

Enterococci are Gram-positive bacteria and are considered the third most common nosocomial pathogens. In Gram-positive bacteria, HPrK/P regulates the transport of carbon source required for growth and other essential functions. It also regulates the phosphorylation of HPr protein, implicated in the virulence processes of pathogenic bacteria. Most importantly, deficiency of HPrK/P reduces the growth of bacteria. Therefore, a search for inhibitors of HPrK/P is of clinical interest. The present study was conducted to identify drug-like compounds able to inhibit the activity of *E. faecalis* HPrK/P. A commercial compound library was computationally screened to identify compounds with high predicted binding energy at an identified site between the P-loop and K3-loop of HPrK/P (named as the ‘K3-loop site'), utilizing a comparative model of *E. faecalis* HprK/P monomer and the structure of a known benzimidazole inhibitor. To evaluate the efficacy of the screened inhibitors, *in-vitro* assays were carried out. Out of the three hit compounds, two (NITSKI8583 and NITSKI5508) potently reduced the activity of HPrK/P and the growth of multi-drug resistant *E. faecalis *in vitro. Putative binding modes of the hit compounds were predicted by molecular docking both at the K3-loop site and at the substrate-binding site of HPrK/P. Subsequent MD simulations of the ligand–protein complexes were carried out to study the stability of the binding interactions, suggesting that the compounds are able to form favorable interactions at the studied sites. However, the predicted binding energies suggest a relatively low affinity of the hit compounds for both the sites, whereas the reference compound clearly prefers the substrate-binding site, consistent with previous research. To be able to confirm the real binding mode and site of the hit compounds, experimental research is required. In conclusion, we have reported potential HPrK/P inhibitor candidates (NITSKI8583 and NITSKI5508) against *E. faecalis*. Further studies are required to verify the compounds’ efficacy on other Gram-positive bacteria to evaluate their usefulness as valuable antimicrobial agents.

## Supplementary Information

Below is the link to the electronic supplementary material.

Supplementary file1 (DOCX 3231 KB)

## Data Availability

The datasets generated during and/or analysed during the current study are available from the corresponding authors.
