# Supplementary material for: Identification of HPr kinase/phosphorylase inhibitors: novel antimicrobials against resistant Enterococcus faecalis
Source: J Comput Aided Mol Des. 2022 Jul 9;36(7):507–20. doi: 10.1007/s10822-022-00461-6 (PMC9399212; doi:10.1007/s10822-022-00461-6)
Supplement: Supplementary file 1 — Supplementary file1 (DOCX 3231 KB) [file 10822_2022_461_MOESM1_ESM.docx]

**Supplementary Information**

**Identification of HPr Kinase/Phosphorylase inhibitors: Novel antimicrobials against resistant *Enterococcus faecalis***

Sandeep Kumar^a^, Rajendra Bhadane^b,c^, Shruti Shandilya^d^, Outi M. H. Salo-Ahen^b,c,*^, Suman Kapila^a,*^

*^a^ Animal Biochemistry Division, National Dairy Research Institute, Karnal, Haryana, India*

*^b^ Structural Bioinformatics Laboratory, Faculty of Science and Engineering, Biochemistry, Åbo Akademi University, FI-20520 Turku, Finland*

*^c^ Pharmaceutical Sciences Laboratory, Faculty of Science and Engineering, Pharmacy, Åbo Akademi University, FI-20520 Turku, Finland*

*^d^ Department of Applied Physics, School of Science, Aalto University, Espoo, Finland*

*Corresponding Authors:

Outi M. H. Salo-Ahen [outi.salo-ahen@abo.fi](mailto:outi.salo-ahen@abo.fi)

Suman Kapila [skapila69@gmail.com](mailto:skapila69@gmail.com)

**Modeller modeling alignment for the full-length monomeric model of *Enterococcus faecalis* HPrK/P**

>P1;1knxA

structureX:1knx_fit.pdb:1 :A:+303 :A: PROBABLE HPR(SER) KINASE/PHOSPHATASE; EC 2.7.1.-, 3.1.3.-: MYCOPLASMA PNEUMONIAE: 2.50: 0.23

MKKLLVKELIEQFQDCVNLIDGHTNTSNVIRVPGLKRVVFEM-LGLFSSQIGSVAILGKREFGFLSQKTLVEQQQ

ILHNLLKLNPPAIILTKSFTDPTVLLQVNQTYQVPILKTDFFSTELSFTVETYINEQFATVAQIHGVLLEVFGVG

VLLTGRSGIGKSECALDLINKNHLFVGDDAIEIYRLG-NRLFGRAQEVAKKFMEIRGLGIINVERFYGLQITKQR

TEIQLMVNLLSL----TFERLGTELKKQRLLGVDLSFYEIPISPGRKTSEIIESAVIDFKLKHSGYNSALDFIEN

QKAILK-RK-K*

>P1;1ko7A

structureX:1ko7_fit.pdb:1 :A:+285 :A: HPR KINASE/PHOSPHATASE; EC 2.7.1.-, 3.1.3.-: STAPHYLOCOCCUS XYLOSUS: 1.95: 0.23

--MLTTKSLVERFE--LEMIAGEAGLNKQIKNTDISRPGLEMAGYFSHYASDRIQLLGTTELSFYNLLPDEERKG

RMRKLCRPETPAIIVTRDLEPPEELIEAAKEHETPLITSKIATTQLMSRLTTFLEHELARTTSLHGVLVDVYGVG

VLITGDSGIGKSETALELIKRGHRLVADDNVEIREISKDELIGRAPKLIEHLLEIRGLGIINVMTLFGAGSILTE

KRLRLNIHLEN-------------EETLRILDTEITKKTIPVRPGRNVAVIIEVAAMNYRLNIMGINTAEEFNDR

LN---------*

>P1;1kklA

structureX:1kkl_fit.pdb:135 :A:+167 :A: HPRK PROTEIN; EC 2.7.1.-, 3.1.3.-: LACTOBACILLUS CASEI: 2.80: 0.21

---------------------------------------------------------------------------

-----------------------------------------------------------ERRSMHGVLVDIYGLG

VLITGDSGVGKSETALELVQRGHRLIADDRVDVYQQDEQTIVGAAPPILSHLLEIRGLGIIDVMNLFGAGAVRED

TTISLIVHLENW----TPD-----EQTQLIFDVPVPKITVPVKVGRNLAIIIEVAAMNFRAKSMGYDATKTFEKN

LNHLIEHNE-E*

>P1;2qmhA

structureX:2qmh_fit.pdb:135 :A:+161 :A: HPR KINASE/PHOSPHORYLASE; EC 2.7.11.-, 2.7.4.-: LACTOBACILLUS CASEI: 2.60: 0.22

---------------------------------------------------------------------------

-----------------------------------------------------------ERRSMHGVLVDIYGLG

VLITGDSGVGKSETALELVQRGHRLIADDRVDVYQQDEQTIVGAAPPILSHLLEIRGLGIIDVMNLFGAGAVRED

TTISLIVHLE--------G-----EQTQLIFDVPVPKITVPFKVGRNLAIIIEVAAMNFRAKSMGYDATKTFEKN

LNHLIEHN---*

>P1;1jb1A

structureX:1jb1_fit.pdb:135 :A:+161 :A: HPRK PROTEIN; EC 2.7.1.-, 3.1.3.-: LACTOBACILLUS CASEI;: 2.80: 0.23

---------------------------------------------------------------------------

-----------------------------------------------------------ERRSMHGVLVDIYGLG

VLITGDSGVGKSETALELVQRGHRLIADDRVDVYQQDEQTIVGAAPPILSHLLEIRGLGIIDVMNLFGAGAVRED

TTISLIVHLENW----TPD-----QLIFD---VPVPKITVPVKVGRNLAIIIEVAAMNFRAKSMGYDATKTFEKN

LNHLIE-H---*

>P1;HprKP

sequence:HprKP: : : : ::: 0.00: 0.00

MTEVVKIYQLVENLSLEVVYGDEESLNRTIKTGEISRPGLELTGYFNYYSHDRLQLFGSKEITFAERMMPEERLL

VMRRLCAKDTPAFIVSRGLEIPEELITAAKENGVSVLRSPISTSRLLGELSSYLDGRLAVRTSVHGVLVDVYGLG

VLIQGDSGIGKSETALELIKRGHRLIADDRVDVYQQDELTVVGEPPKILQHLIEIRGIGIIDVMNLFGASAVRGF

MQVQLVVYLEAWEKDKKYDRLGSDDAMVEIANVDVPQIRIPVKTGRNVAIIIEVAAMNFRAKTMGYDATKTFEER

LTRLIEENSGE*

**Modeller modeling alignment for the dimeric model of *E. faecalis* HPrK/P**

>P1;1kkm_AB

structureX:1kkm_AB.pdb: 135 :A:+353 :B:::-1.00:-1.00

-ERRSMHGVLVDIYGLGVLITGDSGVGKSETALELVQRGHRLIADDRVDVYQQDEQTIVGAAPPILSHLLEIRGL

GIIDVMNLFGAGAVREDTTISLIVHLENWTPDKTFDRLGSGEQTQLIFDVPVPKITVPVKVGRNLAIIIEVAAMN

FRAKSMGYDATKTFEKNLNHLIEHNE-E/E-RRSMHGVLVDIYGLGVLITGDSGVGKSETALELVQRGHRLIADD

RVDVYQQDEQTIVGAAPPILSHLLEIRGLGIIDVMNLFGAGAVREDTTISLIVHLENWTPDKTFDRLGSGEQTQL

IFDVPVPKITVPVKVGRNLAIIIEVAAMNFRAKSMGYDATKTFEKNLNHLIEHNEET*

>P1;HprKP

sequence:HprKP: : : : ::: 0.00: 0.00

AVRTSVHGVLVDVYGLGVLIQGDSGIGKSETALELIKRGHRLIADDRVDVYQQDELTVVGEPPKILQHLIEIRGI

GIIDVMNLFGASAVRGFMQVQLVVYLEAWEKDKKYDRLGSDDAMVEIANVDVPQIRIPVKTGRNVAIIIEVAAMN

FRAKTMGYDATKTFEERLTRLIEENSGE/AVRTSVHGVLVDVYGLGVLIQGDSGIGKSETALELIKRGHRLIADD

RVDVYQQDELTVVGEPPKILQHLIEIRGIGIIDVMNLFGASAVRGFMQVQLVVYLEAWEKDKKYDRLGSDDAMVE

IANVDVPQIRIPVKTGRNVAIIIEVAAMNFRAKTMGYDATKTFEERLTRLIEENSGE*


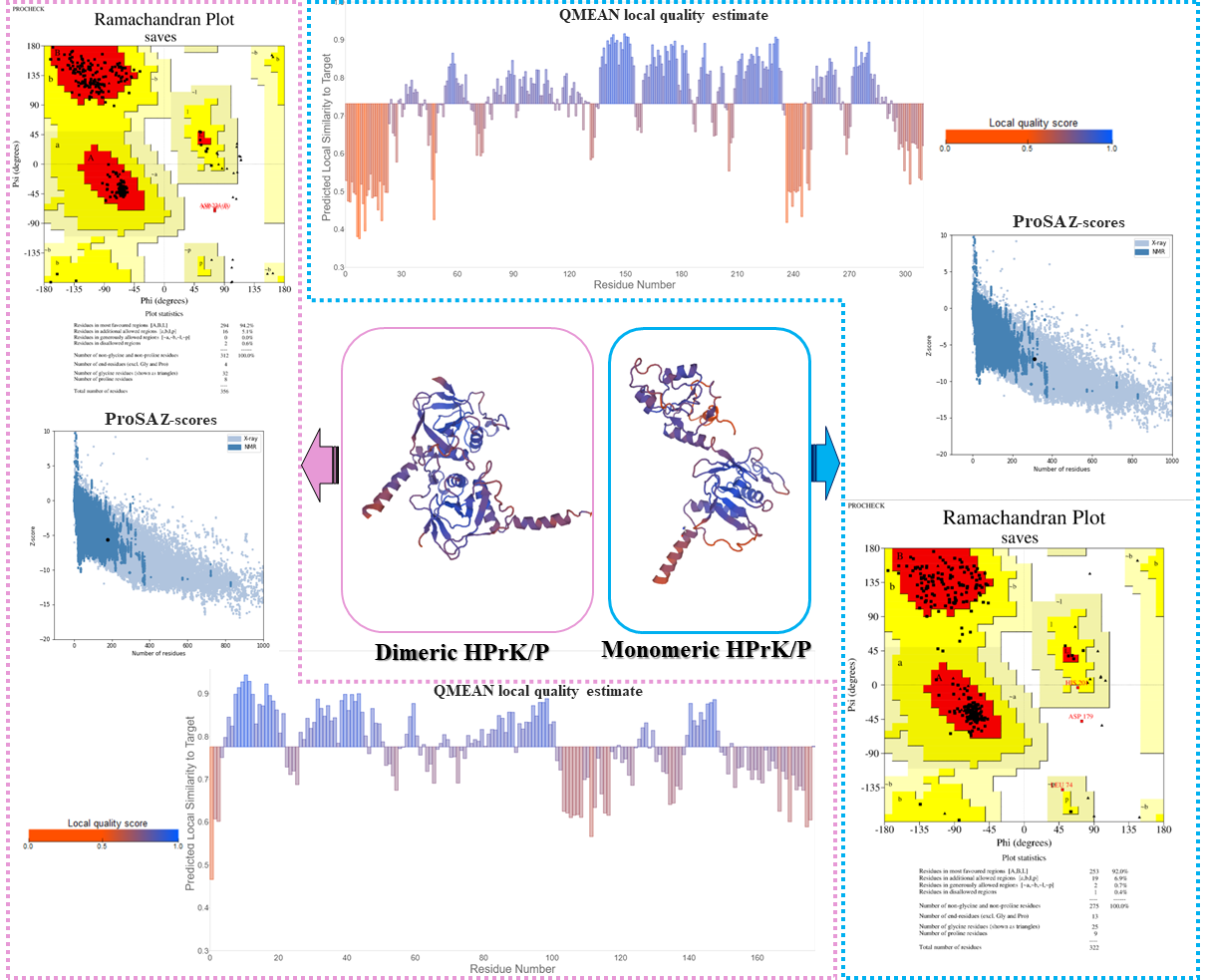


**Figure S1.** Structure validation and quality analysis of the full-length monomeric HPrK/P model and the homodimeric model of the HPrK/P C-terminal catalytic domain. Ramachandran plots were generated on the SAVES server (<https://saves.mbi.ucla.edu>) by PROCHECK. Z-scores were generated by ProSA and the local quality estimate graphs by Qualitative Model Energy Analysis (QMEAN) using the QMEANDisCo method.

**Docking results**


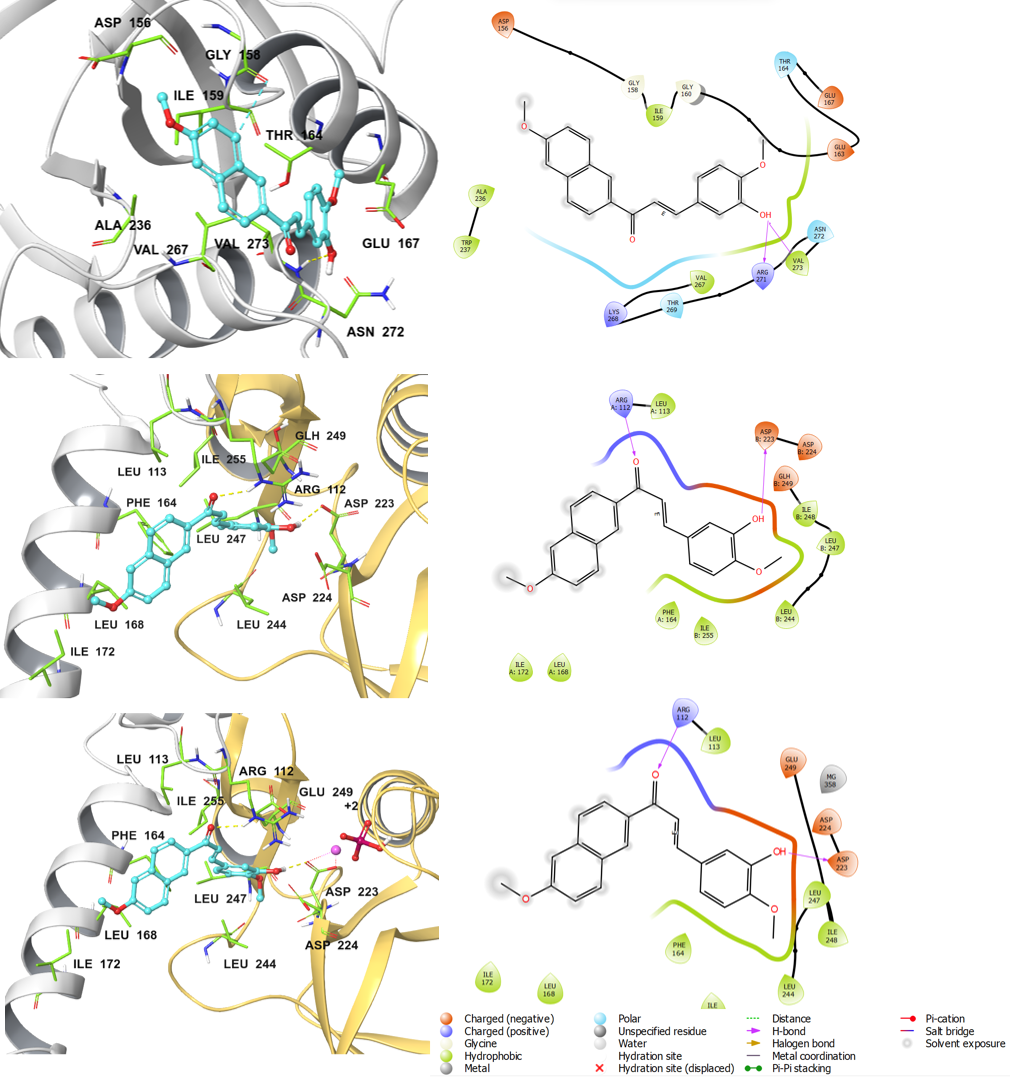


**Figure S2.** The virtual screening hit compound NITSKI152 docked in the HPrK/P substrate-binding site. The docking site interactions are shown at all the three HPrK/P models in 3D (left) and 2D (right). **Top**: full-length monomeric HPrK/P model; Middle: homodimeric model of the HPrK/P C-terminal catalytic domain; **Bottom:** homodimeric model of the HPrK/P C-terminal catalytic domain with Mg^2+^ and PO_4_^3-^ ions. **Left panel:** One monomer chain of HPrK/P is shown in gray color cartoon representation while the other one is in yellow. The docked ligand is shown in ball-and-stick representation (cyan carbon atoms) and the binding site residues are in sticks (green carbon atoms); Mg^2+^ ion is shown as a pink sphere and the phosphate ion in ball-and-stick representation; oxygen atoms are shown in red, nitrogen atoms in blue, phosphorus in dark pink, hydrogen atoms in white. Yellow dashed lines indicate hydrogen bond interactions. Residue numbering in the dimeric models: 1st monomer chain 1-178, 2nd monomer chain 179-356 (corresponding to residues 134-311 in the monomer model).


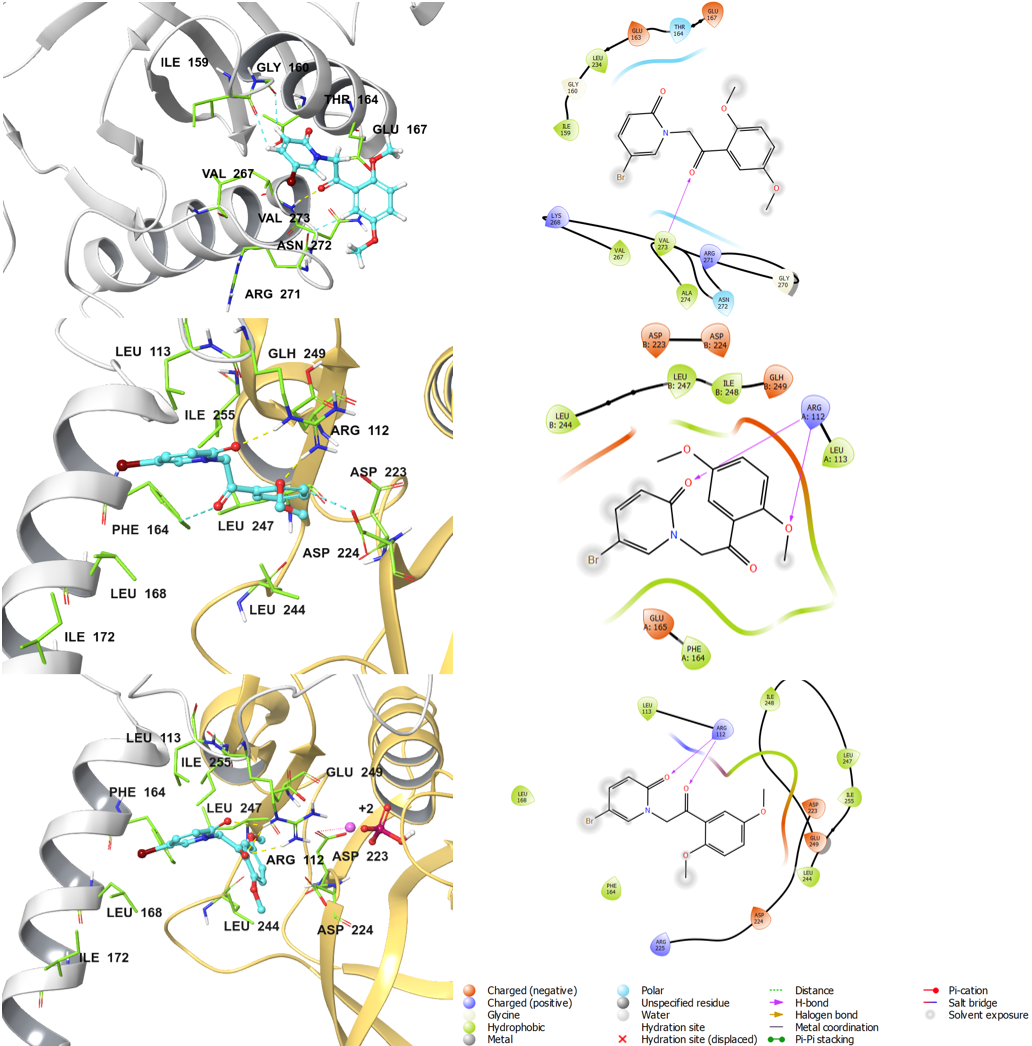


**Figure S3.** The virtual screening hit compound NITSKI8583 docked in the HPrK/P substrate-binding site. The docking site interactions are shown at all the three HPrK/P models in 3D (left) and 2D (right). **Top**: full-length monomeric HPrK/P model; Middle: homodimeric model of the HPrK/P C-terminal catalytic domain; **Bottom:** homodimeric model of the HPrK/P C-terminal catalytic domain with Mg^2+^ and PO_4_^3-^ ions. **Left panel:** One monomer chain of HPrK/P is shown in gray color cartoon representation while the other one is in yellow. The docked ligand is shown in ball-and-stick representation (cyan carbon atoms) and the binding site residues are in sticks (green carbon atoms); Mg^2+^ ion is shown as a pink sphere and the phosphate ion in ball-and-stick representation; oxygen atoms are shown in red, nitrogen atoms in blue, sulphur in yellow, phosphorus in dark pink, hydrogen atoms in white. Yellow dashed lines indicate hydrogen bond interactions. Residue numbering in the dimeric models: 1st monomer chain 1-178, 2nd monomer chain 179-356 (corresponding to residues 134-311 in the monomer model).

MD results – Stability of the ligand-protein complexes

*Full-length monomeric* ***model of E. faecalis*** *HPrK/P*

**Figure S4.** Backbone root mean square deviation (RMSD) of the full-length monomeric HPrK/P model with the docked hit compounds during a 100-ns molecule dynamics (MD) simulation (each compound was simulated in three replicates and the trajectory was averaged).

**Figure S5.** Backbone root mean square fluctuation (RMSF) of the residues in the full-length monomeric HPrK/P model with the docked hit compounds during a 100-ns molecule dynamics (MD) simulation. N-terminal domain: residues 1-134; C-terminal catalytic domain: res. 134-311; flexible/disordered loop: res. 235-251 (each compound was simulated in three replicates and the trajectory was averaged).

*Homodimeric model of the E. faecalis HPrK/P C-terminal catalytic domain*

**Figure S6.** Backbone root mean square deviation (RMSD) of the homodimeric model of the HPrK/P C-terminal catalytic domain with the docked hit compounds during a 100-ns molecule dynamics (MD) simulation (each compound was simulated in three replicates and the trajectory was averaged).

**Figure S7.** Backbone root mean square fluctuation (RMSF) of the residues in the homodimeric model of the HPrK/P C-terminal catalytic domain with the docked hit compounds during a 100-ns molecule dynamics (MD) simulation. Residue numbering: 1^st^ monomer chain 1-178, 2^nd^ monomer chain 179-356 (corresponding to residues 134-311 in the monomer model); flexible/disordered loop: res. 235-251 (102-118 in the 1^st^ chain; 280-296 in the 2^nd^ chain) (each compound was simulated in three replicates and the trajectory was averaged).

*Homodimeric model of the E. faecalis HPrK/P C-terminal catalytic domain (with ions)*

**Figure S8.** Backbone root mean square deviation (RMSD) of the homodimeric model of the HPrK/P C-terminal catalytic domain (with Mg^2+^ and PO_4_^3-^ ions) with the docked hit compounds during a 100-ns molecule dynamics (MD) (each compound was simulated in three replicates and the trajectory was averaged).

**Figure S9.** Backbone root mean square fluctuation (RMSF) of the residues in the homodimeric model of the HPrK/P C-terminal catalytic domain (with Mg^2+^ and PO_4_^3-^ ions) with the docked hit compounds during a 100-ns molecule dynamics (MD) simulation. Residue numbering: 1^st^ monomer chain 1-178, 2^nd^ monomer chain 179-356 (corresponding to residues 134-311 in the monomer model); flexible/disordered loop: res. 235-251 (102-118 in the 1^st^ chain; 280-296 in the 2^nd^ chain) (each compound was simulated in three replicates and the trajectory was averaged).

**
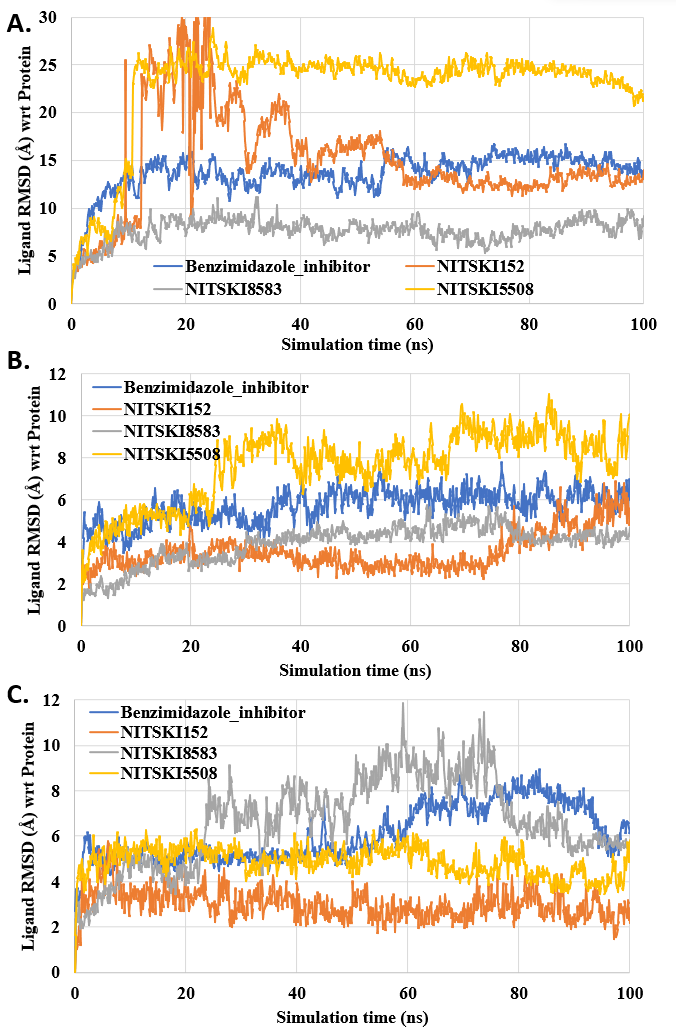
**

**Figure S10.** Ligand RMSD – Fluctuations of the hit compounds with respect to (wrt) the HPrK/P protein model during a 100-ns molecule dynamics (MD) simulation. **A.** full-length monomeric HPrK/P model; **B.** homodimeric model of the HPrK/P C-terminal catalytic domain; **C.** homodimeric model of the HPrK/P C-terminal catalytic domain with Mg^2+^ and PO_4_^3-^ ions.

**Table S1.** Ligand-HPrK/P interactions during the MD simulations

| **Compound** | ***E. faecalis* HPrK/P model** | **Residues participating in H-bond interactions*** | **Residues participating in hydrophobic interactions*** |
| --- | --- | --- | --- |
| Benzimidazole inhibitor 3-B-3 | Monomeric | **Ile159**, Glu163, Lys239, Thr269 | **Ile159**, **Ala236**, Lys239, **Val267**, **Val273** |
|  | Dimeric | Glu174 (Glu307), Asn175 (Asn308), Glu178 (311), Asp224’ (**Asp179’**) | Arg112 (**Arg245**), Phe164 (**Phe297**), Leu168 (**Leu301**), Leu171 (Leu304), Leu244’ (**Leu199’**), Ile 255’ (**Ile210’**) |
|  | Dimeric with PO_4_^3-^ and Mg^2+^ | Asp224’ (**Asp179’**), Asp227’ (Asp182’), Lys242’ (Lys197’) | Arg112 (**Arg245**), Phe164 (**Phe297**), Leu168 (**Leu301**), Leu171 (Leu304), Arg225’ (Arg180’), Lys242’ (Lys197’), Ile 255’ (**Ile210’**) |
| NITSKI152 | Monomeric | Thr164, Arg271 | **Ala236**, Trp237, **Val267**, **Val273** |
|  | Dimeric | Asp223’ (**Asp178’**) | Leu113 (**Leu246**), Phe164 (**Phe297**), Leu168 (**Leu301**), Leu244’ (**Leu199’**), Ile255’ (**Ile210’**) |
|  | Dimeric with PO_4_^3-^ and Mg^2+^ | Arg112 (**Arg245**), Asp223’ (**Asp178’**) | Leu113 (**Leu246**), Phe164 (**Phe297**), Leu168 (**Leu301**), Leu244’ (**Leu199’**), Ile255’ (**Ile210’**) |
| NITSKI8583 | Monomeric | Asn272, **Val273** | **Val273** |
|  | Dimeric | Arg112 (**Arg245**) | Leu113 (**Leu246**), Phe164 (**Phe297**), Leu244’ (**Leu199’**) |
|  | Dimeric with PO_4_^3-^ and Mg^2+^ | Arg112 (**Arg245**) | Phe164 (**Phe297**), Leu168 (**Leu301**), Leu244’ (**Leu199’**), Ile 255’ (**Ile210’**) |
| NITSKI5508 | Monomeric | **Ile159**, **Val273** | **Val267**, **Val273** |
|  | Dimeric | Arg112 (**Arg245**), Asp223’ **(Asp178)** | Arg112 (**Arg245**), Phe164 (**Phe297**), Leu244’ (**Leu199’**), Ile255’ (**Ile210’**) |
|  | Dimeric with PO_4_^3-^ and Mg^2+^ | Arg112 (**Arg245**), Asp223’ (**Asp178’**) | Arg112 (**Arg245**), Phe164 (**Phe297**), Leu244’ (**Leu199’**), Leu247’ (**Leu202’**), Ile255’ (**Ile210’**) |

* Residues denoted with a prime (‘) are from the second chain of the dimeric model; residue numbering in the dimeric models: 1^st^ monomer chain 1-178, 2^nd^ monomer chain 179-356 (corresponding residue number in the monomeric model in the brackets); flexible/disordered loop: res. 235-251 (dimer: 102-118 in the 1^st^ chain; 280-296 in the 2^nd^ chain). Residues in bold denote amino acids in the monomeric and/or dimeric models that interact with more than one ligand.

*
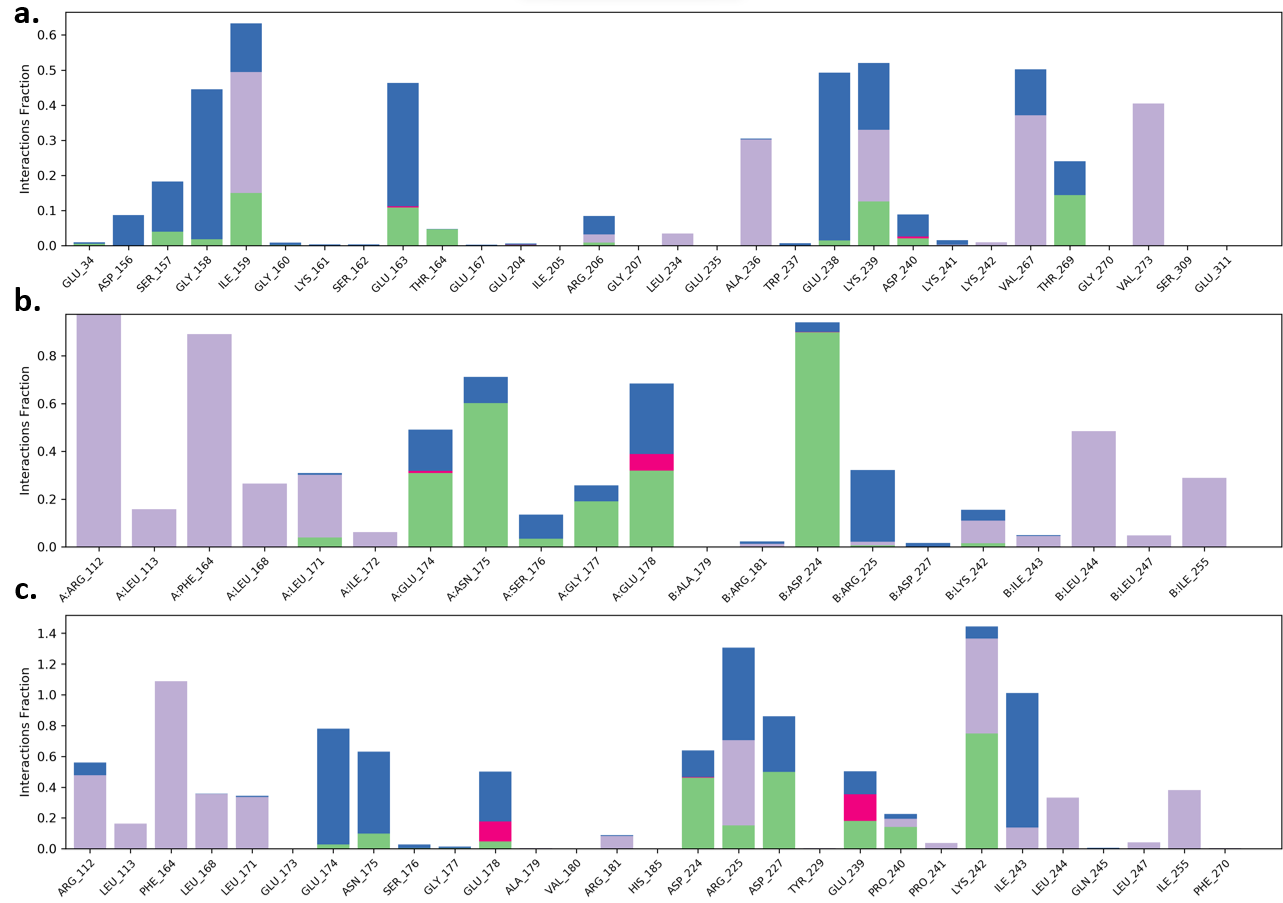
*

**Figure S11: Protein-ligand contacts.** HPrK/P interactions with the reference benzimidazole inhibitor monitored throughout the 100-ns molecular dynamics (MD) simulations. **(a)** Monomeric full length HPrK/P model; **(b)** homodimeric C-terminal domain model of HPrK/P; **(c)** homodimeric C-terminal domain model of HPrK/P with PO_4_^3-^ and Mg^2+^. Residue numbering in the dimeric model: 1-178 (1^st^ subunit); 179-356 (2^nd^ subunit) corresponds the C-terminal domain residue range 134-311. Color code for interactions: purple – hydrophobic interaction; green – hydrogen bonding; blue – water mediated interaction; dark pink – electrostatic interaction (i.e. salt bridge, metallic bond). If the interactions fraction exceeds 1.0 (100% of the simulation time), the respective residue forms more than one interaction with the ligand.


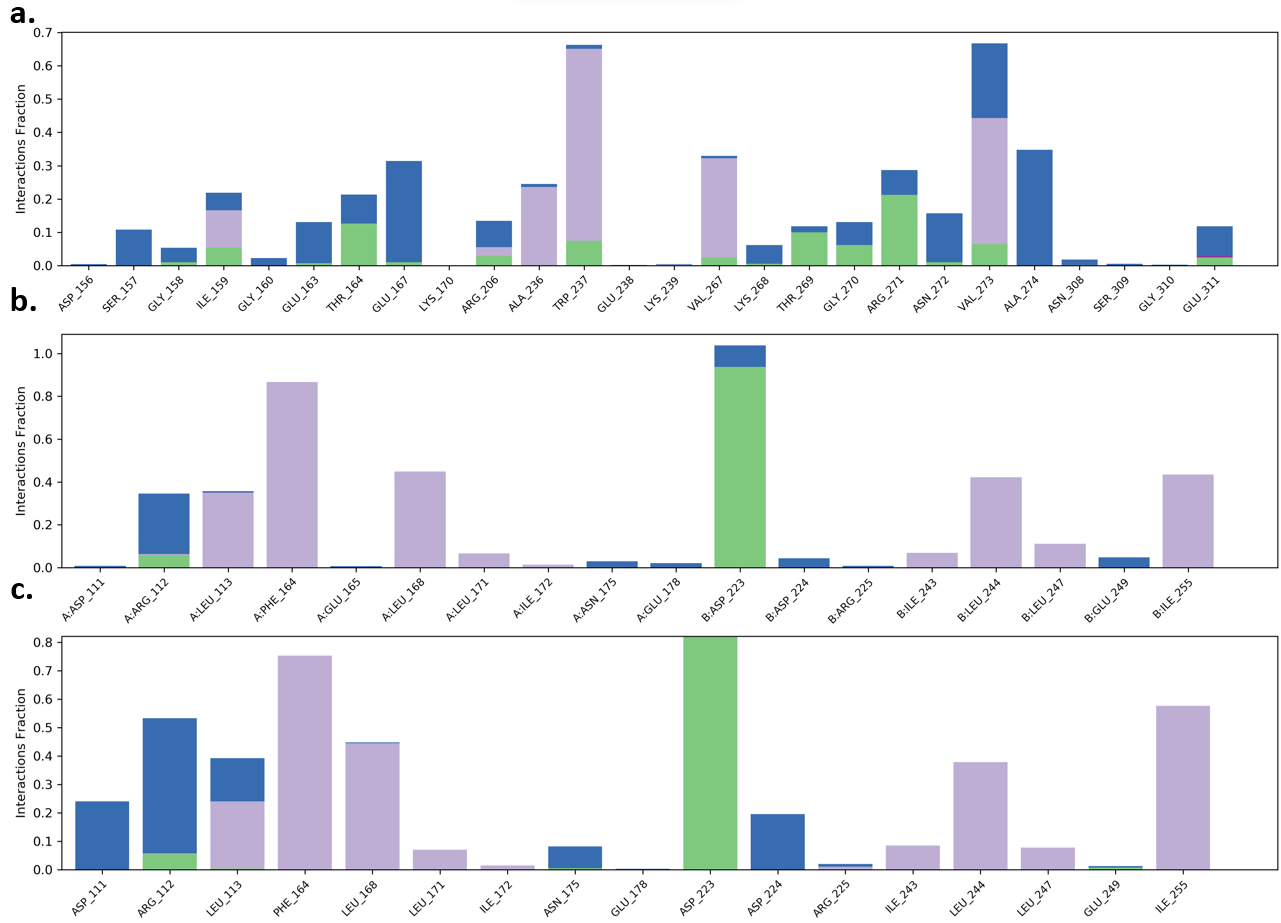


**Figure S12: Protein-ligand contacts.** HPrK/P interactions with the hit compound NITSKI152 monitored throughout the 100-ns molecular dynamics (MD) simulations. **(a)** Monomeric full length HPrK/P model; **(b)** homodimeric C-terminal domain model of HPrK/P; **(c)** homodimeric C-terminal domain model of HPrK/P with PO_4_^3-^ and Mg^2+^. Residue numbering in the dimeric model: 1-178 (1^st^ subunit); 179-356 (2^nd^ subunit) corresponds to the C-terminal domain residue range 134-311. Color code for interactions: purple – hydrophobic interaction; green – hydrogen bonding; blue – water mediated interaction; dark pink – electrostatic interaction (i.e. salt bridge, metallic bond). If the interactions fraction exceeds 1.0 (100% of the simulation time), the respective residue forms more than one interaction with the ligand.


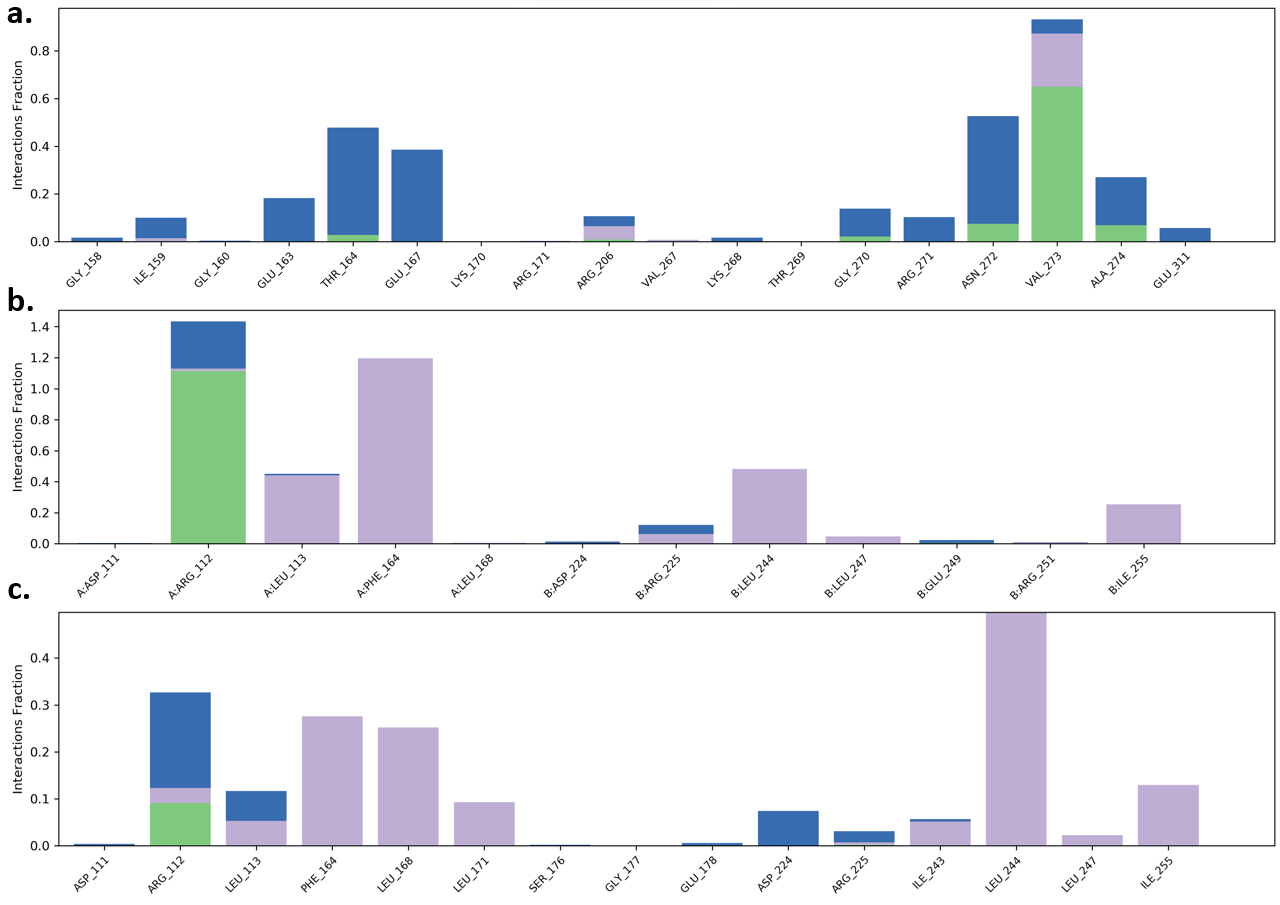


**Figure S13: Protein-ligand contacts.** HPrK/P interactions with the hit compound NITSKI8583 monitored throughout the 100-ns molecular dynamics (MD) simulations. **(a)** Monomeric full length HPrK/P model; **(b)** homodimeric C-terminal domain model of HPrK/P; **(c)** homodimeric C-terminal domain model of HPrK/P with PO_4_^3-^ and Mg^2+^. Residue numbering in the dimeric model: 1-178 (A chain); 179-356 (B chain) corresponds the C-terminal domain residue range 134-311. Color code for interactions: purple – hydrophobic interaction; green – hydrogen bonding; blue – water mediated interaction; dark pink – electrostatic interaction (i.e. salt bridge, metallic bond). If the interactions fraction exceeds 1.0 (100% of the simulation time), the respective residue forms more than one interaction with the ligand.


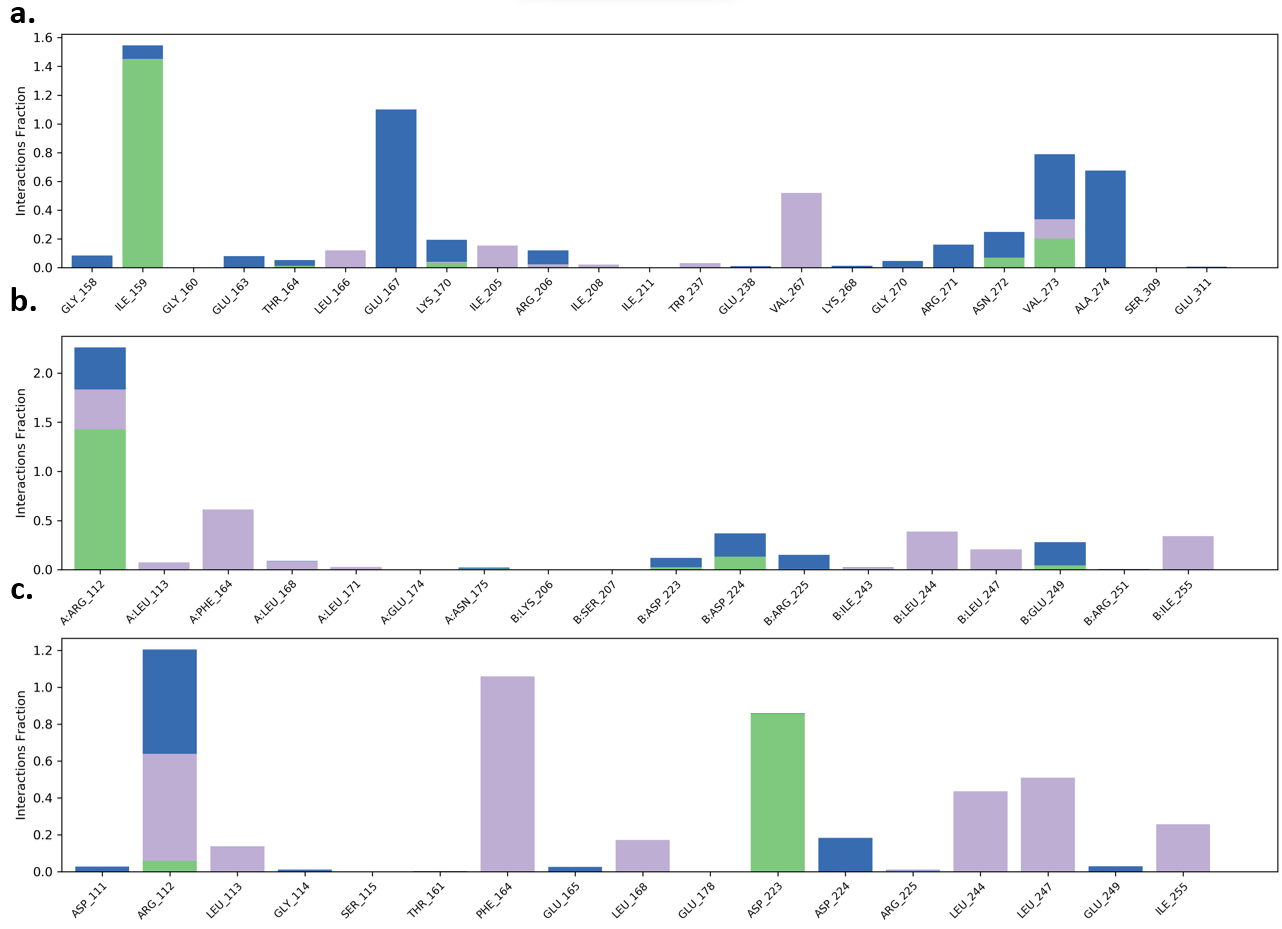


**Figure S14: Protein-ligand contacts.** HPrK/P interactions with the hit compound NITSKI5508 monitored throughout the 100-ns molecular dynamics (MD) simulations. **(a)** Monomeric full length HPrK/P model; **(b)** homodimeric C-terminal domain model of HPrK/P; **(c)** homodimeric C-terminal domain model of HPrK/P with PO_4_^3-^ and Mg^2+^. Residue numbering in the dimeric model: 1-178 (1st subunit); 179-356 (2nd subunit) corresponds the C-terminal domain residue range 134-311. Color code for interactions: purple – hydrophobic interaction; green – hydrogen bonding; blue – water mediated interaction; dark pink – electrostatic interaction (i.e. salt bridge, metallic bond). If the interactions fraction exceeds 1.0 (100% of the simulation time), the respective residue forms more than one interaction with the ligand.


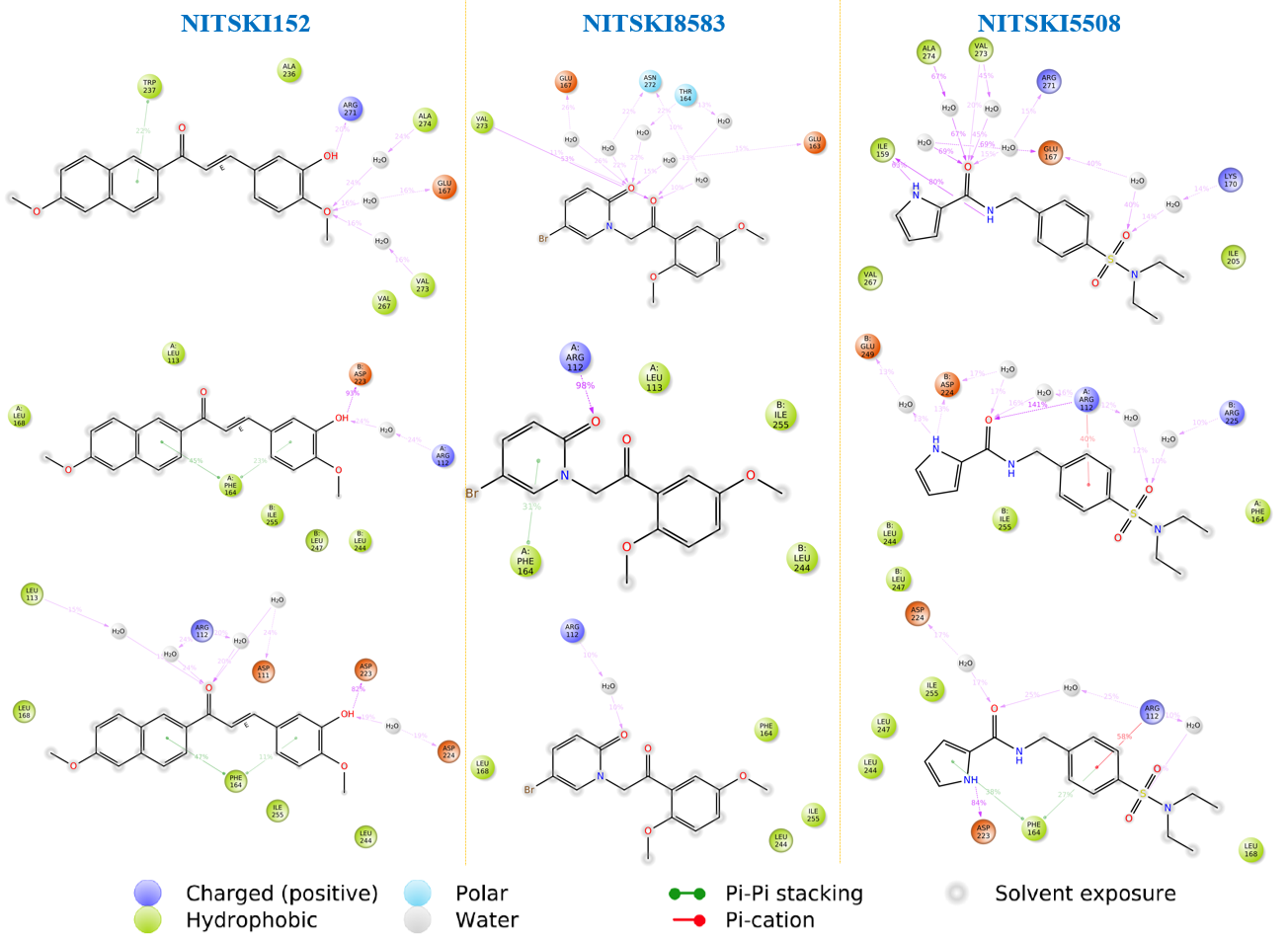


**Figure S15.** 2D ligand interaction plots generated after the 100-ns MD simulation of the docked ligands in the HPrK/P models. The interactions that lasted for more than 5% of the simulation time are shown. **Top**: full-length monomeric HPrK/P model; **Middle:** homodimeric model of the HPrK/P C-terminal catalytic domain; **Bottom:** homodimeric model of the HPrK/P C-terminal catalytic domain with Mg^2+^ and PO_4_^3-^ ions. Colour scheme: pink arrows – hydrogen bond interaction; red lines – aromatic hydrogen bond interaction; green lines – pi-pi aromatic stacking interaction. Residue numbering in the dimeric model: 1-178 (1st subunit); 179-356 (2nd subunit) corresponds the C-terminal domain residue range 134-311.


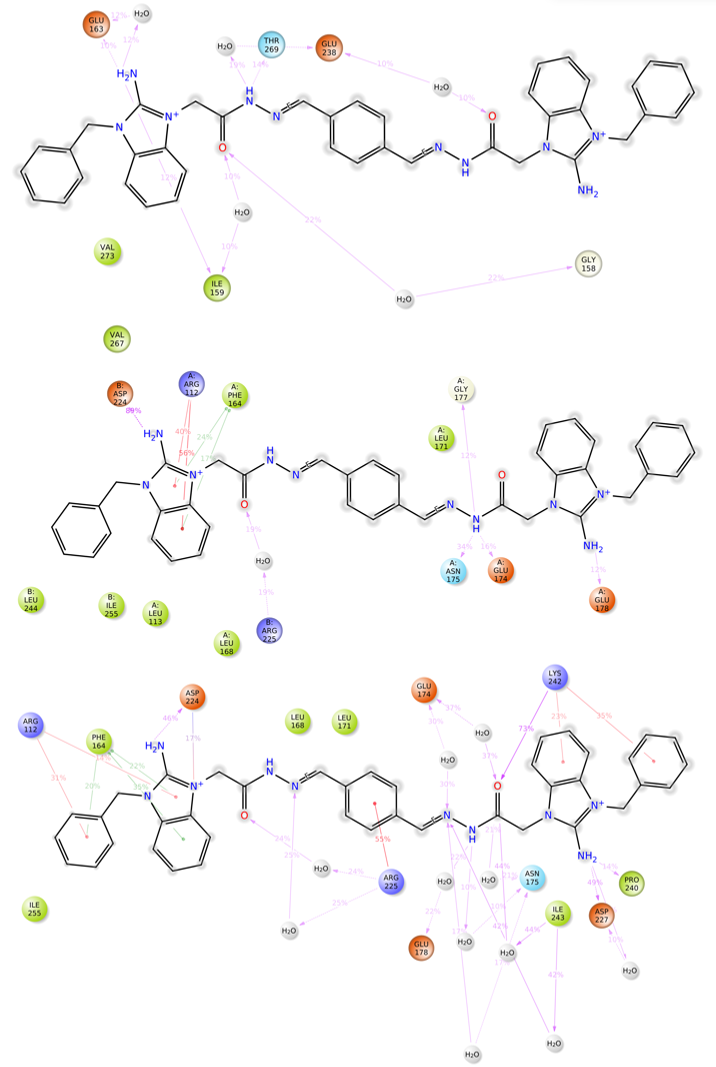


**Figure S16.** 2D ligand interaction plots generated after the 100-ns MD simulation of the benzimidazole inhibitor in the HPrK/P models. The interactions that lasted for more than 10% of the simulation time are shown. **Top**: full-length monomeric HPrK/P model; **Middle:** homodimeric model of the HPrK/P C-terminal catalytic domain; **Bottom:** homodimeric model of the HPrK/P C-terminal catalytic domain with Mg^2+^ and PO_4_^3-^ ions. Colour scheme: pink arrows – hydrogen bond interaction; red lines – aromatic hydrogen bond interaction; green lines – pi-pi aromatic stacking interaction. Residue numbering in the dimeric model: 1-178 (1st subunit); 179-356 (2nd subunit) corresponds the C-terminal domain residue range 134-311.


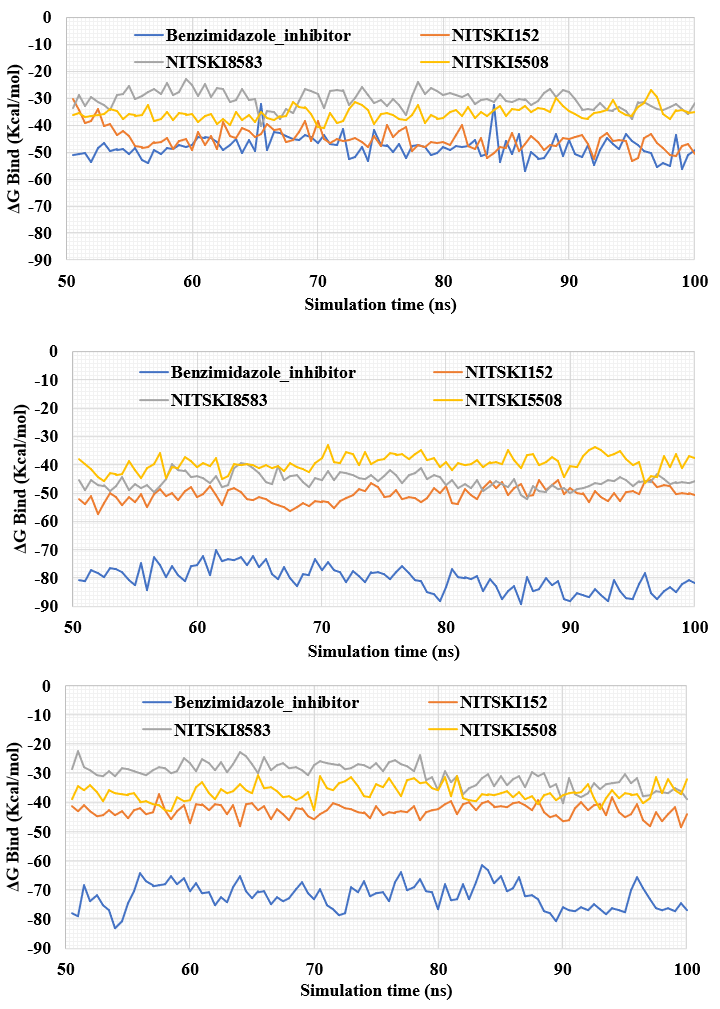


**Figure S17.** Prime/MM-GBSA binding free energy estimation for the docked virtual screening hit compounds at the monomeric HPrK/P model (top) and the substrate binding site of the homodimeric HPrK/P model (middle) and homodimeric HPrK/P model with ions (bottom) during the last 50 ns of the MD simulation (for each compound the binding free energy is estimated from three independent MD simulations and averaged).

**Decoy docking:**

In an attempt to evaluate if the hit compounds were rather found by serendipity than by using the search criteria based on the selected site and the reference compound, we performed a simple decoy docking procedure using a Knime virtual screening workflow.^[[1]](#footnote-1)^ To generate a decoy dataset, we first calculated molecular properties of the three hit compounds (molecular weight, number of hydrogen bond donors and acceptors, logP, number of rotatable bonds) employing the RDKit descriptor calculator (RDKit: Open-source cheminformatics; <https://www.rdkit.org>). In the same way, we calculated these properties for a database of more than 1 million compounds from Molport (<https://www.molport.com>). The database compounds were then filtered by these properties to select only the compounds with closely similar properties to the hit compounds (MW ± 50 Da, logP ± 0.5, H-bond donors/acceptors ± 1, rotatable bonds ± 2). Thereafter, the RDKit fingerprint generator was used to generate 1024 hashed bit-based Daylight-like topological fingerprints for the hits and the database compounds. A similarity search was then carried out by comparing the fingerprints of the hit compounds and those of the database compounds. To find dissimilar compounds to the hit compounds as decoys, the Tanimoto similarity index of < 0.32 was used as the result-limiting criteria. In this way, a total of 386 decoys with similarity index between 0.22 and 0.32 were obtained. The decoys and the hit molecules were then prepared by LigPrep and docked to the ‘K3-loop site’ of the monomeric model and the substrate binding site of the dimeric models using the same Glide docking protocol as used for the binding mode analysis (see Methods). The Glide docking scores obtained were then used to generate the receiver operating characteristic (ROC) curve and the area under curve (AUC) was determined (Figure S18).


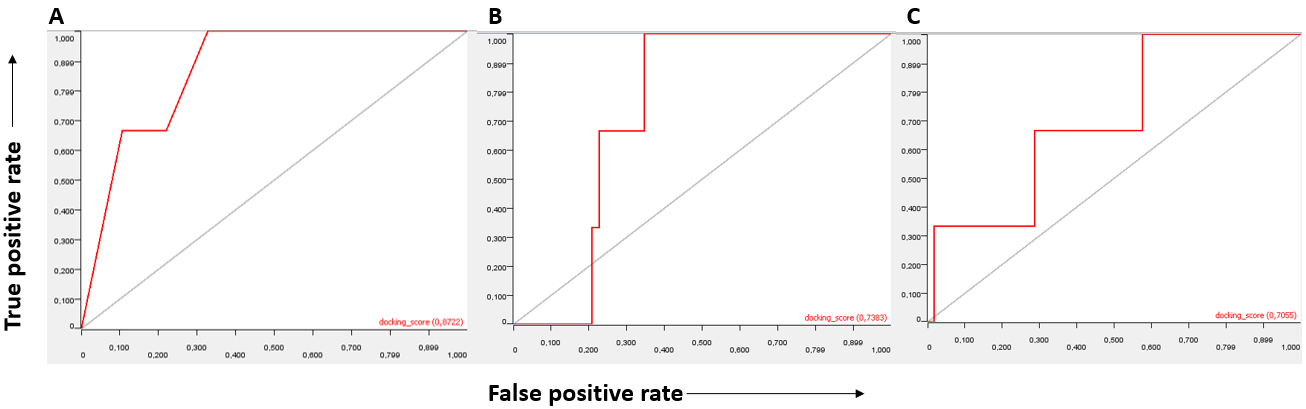


**Figure 18.** The Receiver Operating Characteristic (ROC) curves from the decoy docking. **A.** ROC curve for the decoy docking at the K3-loop site of the full-length monomeric HPrK/P model with an Area Under Curve (AUC) of 0.8722 using the Glide docking score. **B.** ROC curve for the decoy docking at the substrate-binding site of the dimeric model of the HPrK/P C-terminal catalytic domain with AUC of 0.7383 using the Glide docking score. **C.** ROC curve for the decoy docking at the substrate-binding site of the dimeric model of the HPrK/P C-terminal catalytic domain with Mg^2+^ and PO_4_^3-^ ions with AUC of 0.7055 using the Glide docking score.

Docking to the ‘K3-loop site’ suggests a better performance in enriching the actives than the substrate-binding site, which would be consistent with the fact that this site was used for the initial virtual screening. However, this approach is limited by the fact that there were only three actives in the dataset, and we do not know with certainty that all decoys are inactive.

1. Berthold, M.R. et al. (2008). KNIME: The Konstanz Information Miner. In: Preisach, C., Burkhardt, H., Schmidt-Thieme, L., Decker, R. (eds) Data Analysis, Machine Learning and Applications. Studies in Classification, Data Analysis, and Knowledge Organization. Springer, Berlin, Heidelberg. [↑](#footnote-ref-1)
